# Repositioning of Alogliptin to Mitigate Secondary Injury Induced by Repetitive TBI: Potential Role of its Antioxidant and Anti- Inflammatory Effects

**DOI:** 10.1007/s11481-025-10271-w

**Published:** 2026-01-13

**Authors:** Hossam A. Raslan, Haidy E. Michel, Esther T. Menze, Amira A. El-Gazar

**Affiliations:** 1 Department of Pharmacology & Toxicology, Faculty of Pharmacy, October 6 University, Giza, Egypt; 2 Department of Pharmacology and Toxicology, Faculty of Pharmacy, Ain Shams University, Cairo, 11566 Egypt

**Keywords:** Alogliptin, RTBI, Endoplasmic reticulum stress, MiRNA-322, MiRNA-125b

## Abstract

Repetitive traumatic brain injury (RTBI) refers to brain injuries resulting from an external mechanical force causing cumulative and frequently severe neurological consequences. This study aimed to explore the neuroprotective effect of alogliptin (ALO) on RTBI-provoked endoplasmic reticulum (ER) stress and investigate the potential underlying mechanisms. For RTBI induction, rats were exposed to a sharp-edged weight at the right interior frontal area of the right cortex, one drop per day for five successive days. ALO (20 mg/kg/day, p.o.) was administered for one week. Results depicted that ALO recovered motor abnormalities and enhanced motor coordination in the open field test, decreased immobility and increased climbing time in the forced swimming test, and corrected histological aberrations. Moreover, ALO counteracted RTBI-triggered ER stress via suppression of activating transcription factor 6 (ATF6), glucose-regulated protein 78 (GRP78), aggregation of β-amyloid and Tau proteins, as well as elevation of the cortical content of brain-derived neurotrophic factor (BDNF) and its receptor, tropomyosin receptor kinase B (TrKB). ALO also exhibited an antioxidant and anti-inflammatory potential in addition to its effect on the gene expression of miRNAs (miRNA-322 and miRNA-125b). In conclusion, ALO exhibited a neuroprotective effect by mitigating ER stress induced in an RTBI rat model.

**Graphical Abstract:**

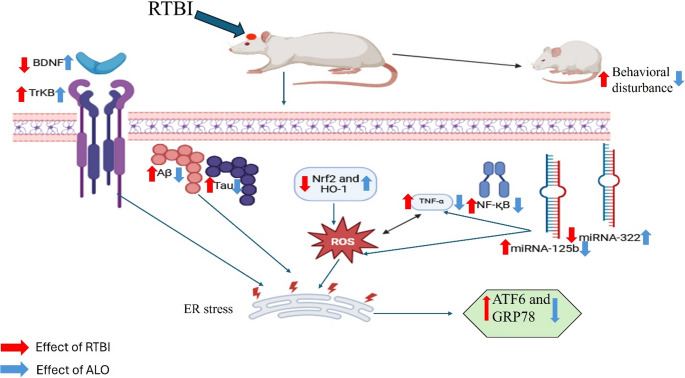

**Supplementary Information:**

The online version contains supplementary material available at 10.1007/s11481-025-10271-w.

## Introduction

Repetitive traumatic brain injury (RTBI) is a progressive neurodegenerative disorder affecting individuals subjected to repeated head trauma. Mostly, repeated mild RTBI incidents can lead to a more pernicious, long-lasting condition in the brain (Guskiewicz et al. 2003). Beta-amyloid (Aβ) deposition can be facilitated by single and RTBI, which may increase the risk of neurodegenerative disorders, such as Alzheimer’s disease (AD) (Ramos-Cejudo et al. 2018; Ojo et al. 2019), Parkinson’s disease (PD) (Delic et al. 2020; Edwards et al. 2025), and amyotrophic lateral sclerosis (Majewski et al. 2012; Gu et al. 2021). The building up of hyperphosphorylated Aβ or tau proteins may initiate irreversible endoplasmic reticulum (ER) stress, leading to synaptic dysfunction and neurodegeneration (Duran-Aniotz et al. 2014).

ER stress has a marked role in secondary neuronal damage after TBI, leading to various pathological and behavioral deficits (Tan et al. 2018). Physiologically, the ER coordinates the correct folding and sorting of proteins and accumulates unfolded and misfolded ER proteins through three major ER stress sensors mediating the unfolded protein response (UPR) to reduce ER stress by activating multiple biochemical processes to prevent this scenario and restore homeostasis (Nakka et al. 2010; Tsai and Weissman 2010). Activating transcription factor 6 (ATF6) is one of the ER stress sensors (Sano and Reed 2013; Li et al. 2014) that regulates UPR by interaction with glucose-regulated protein 78 (GRP78) (Lee 2001). ATF6 decreases protein production and its entry into the ER. Moreover, ATF6 regulates antioxidant defense signaling pathways (Jin et al. 2017). One of the antioxidant systems that affects ER stress is nuclear factor erythroid 2-related factor 2 (Nrf2), and vice versa, as ER/UPR signals can attenuate Nrf2 gene expression and its downstream molecules. Nrf2 activates a number of antioxidant enzymes, including heme oxygenase-1 (HO-1), that removes reactive oxygen species (ROS) and other harmful substances, and it helps to prevent oxidative stress, inflammation, and apoptosis (Zhao et al. 2017; Cui et al. 2019). The brain-derived neurotrophic factor **(**BDNF) is a neurotrophic factor that improves neuronal function in neurodegenerative diseases (Benarroch 2015). One of the neuroprotective mechanisms of BDNF is supposed to be through suppressing C/EBP homologous protein (CHOP) production, one of the ER stress mediators (Rodrigues and Ballesteros 2007).

MicroRNAs are short non-coding RNAs that have a crucial role in post-transcriptional modulation of gene expression (Ha and Kim 2014). MicroRNAs have been extensively documented as possible biomarkers for neurodegenerative disorders (Hayes et al. 2014; Ma et al. 2017; Zhang et al. 2019; Liu et al. 2020; Bahlakeh et al. 2021). Hence, this study design included miRNA (322&125b) tracking and elucidating their role in secondary brain damage caused by RTBI. The selection of both miRNAs depended on their documented role in brain disorders, especially neurodegenerative ones, as well as their post-transcriptional regulation of key pathological processes such as neuroinflammation, oxidative stress, and death signaling (Parisi et al. 2016; Zhang et al. 2021; Zheng et al. 2022). miRNA-322 exhibited a protective effect in a chronic brain hypoperfusion model, where the overexpression of miRNA-322-5p decreased neuronal death and inflammation while improving the memory function (Zheng et al. 2022). Moreover, miRNA-322-5p markedly suppressed TLR4/TRAF6/NF-κB mediated inflammation and decreased neuronal death (Zhou et al. 2022) in an experimental model of epilepsy. Overexpression of miRNA-125b in primary hippocampal neurons exacerbated the pathogenesis of AD by increasing Tau phosphorylation, which lead to neuronal death (Banzhaf-Strathmann et al. 2014; Zhang et al. 2019). miRNA-125b was documented as a potential biomarker of AD (Cogswell et al. 2008). In addition, it induces an inflammatory response through activation of NF-қB (Zhang et al. 2021).

Repositioning of gliptins, a class of drugs primarily used to treat type 2 diabetes by inhibiting the activity of dipeptidyl peptidase-4 (DPP-4), is being investigated for their potential neuroprotective effects. Gliptins increase the levels of incretin by inhibiting the activity of dipeptidyl peptidase-4 (DPP-4), an enzyme responsible for breaking down glucagon-like peptide-1 (GLP-1) (Srinivasan et al. 2008). GLP-1 not only crosses the blood-brain barrier but is also synthesized within the brain, where it interacts with GLP-1 receptors to exert anti-inflammatory and anti-apoptotic effects, thereby protecting against neurodegeneration (Hölscher 2014). Alogliptin (ALO) is a strong and highly selective inhibitor of DPP-4 (Feng et al. 2008; Moritoh et al. 2008). By inhibiting DPP-4, ALO elevates GLP-1 levels, resulting in anti-inflammatory properties and enhancing synaptic plasticity (Rahman et al. 2020; El-Sahar et al. 2021; Safar et al. 2021). ALO has also been shown to reduce the accumulation of Aβ fibrils and plaque deposition (Rahman et al. 2020), a key pathological feature of AD. Mechanistically, ALO suppresses the activation of nuclear factor kappa B (NFκB), thereby attenuating the expression of downstream pro-inflammatory cytokines such as tumor necrosis factor-alpha (TNF-α) (Botros et al. 2024; El-Sayed et al. 2024). In parallel, ALO upregulates the nuclear factor erythroid 2-related factor 2/heme oxygenase-1 (Nrf2/HO-1) pathway, enhancing the cellular antioxidant response (El-Sayed et al. 2024). Furthermore, ALO exhibited a remarkable neuroprotective effect in normal non-diabetic mice with localized cerebral ischemia (Yang et al. 2013).

Hence, this study aimed to reveal the crosstalk between ER stress and RTBI and its role in secondary brain damage and behavioral dysfunction associated with RTBI. Besides, the potential neuroprotective capacity of ALO against RTBI was investigated by tracking ER stress markers and the interconnection with BDNF/TrKB, inflammatory processes, oxidative stress, as well as the post-transcriptional modulation of miRNAs (322 and 125b).

## Materials and Methods

### Animal Care and Ethics Statement

Male adult Sprague-Dawley (SD) rats (*n* = 36) weighing 250±20 gm were procured from The Egyptian Company for the Production of Serums, Vaccines, and Drugs (Helwan, Egypt). Rats were left to acclimatize for one week to the new ambiance (temperature of 22 ± 2 °C, humidity of 55% ± 5%, 12-h light/dark cycle, and automatically controlled ventilation) before experimentation. A standard chow meal and unlimited amounts of water were provided.

In this study, the resource equation was used to determine the sample size (Arifin and Zahiruddin 2017). Minimum sample size = (10/k) + 1, and maximum sample size = (20/k) + 1, where k = number of groups, were calculated. Accordingly, the number of animals per group ranged from 4 to 6. To account for potential animal loss and to fulfill the different analyses previously designed, we increased the sample size to 9 rats per group.

### Drugs and Chemicals

Alogliptin was purchased from EVA Pharmaceutical Company in Giza, Egypt.

### Experimental Design

Animals were distributed following a simple randomization method (Aldeeb et al. 2024) into four groups (*n* = 9). Animals in group 1 served as a control that underwent the same handling and anesthesia procedures as the RTBI groups and received 1% tween 80 (the vehicle of alogliptin) (Safar et al. 2021), whereas those in group 2 were treated with alogliptin (ALO; 20 mg/kg, p.o.) (Akita et al. 2015; Kabel 2018) to serve as an alogliptin control. Animals in groups 3 and 4 received one blow for five consecutive days to provoke RTBI. Group 3 was left without any intervention and marked as RTBI, whereas group 4 animals received ALO (20 mg/kg, p.o.) for one week starting from the day of the last blow and were signified as RTBI + ALO.

### Induction of Repetitive Trauma

To mimic normal TBI/RTBI in highly susceptible individuals (Costanza et al. 2011), closed-head injury was provoked using a weight drop device. Briefly, A 75 g metallic cylindrical weight with a sharp conical tip (tip diameter approximately 1–2 mm, cone angle ~ 15–20°) (El-Gazar et al. 2019, 2024b), as shown in Fig. of weight used in the supplementary file, was allowed to freely fall through a vertical guiding tube from a height of 25 cm to deliver an impact energy of approximately 0.5 J to the skull. The impact was targeted to the right interior frontal cortex, corresponding anatomically to 2–3 mm lateral to the midline and 1–2 mm anterior to bregma (Albert-Weißenberger et al. 2012). All animals were anesthetized using isoflurane, using 4% isoflurane and placed on the platform directly under the weight drop device. Anesthesia was maintained by mask inhalation of isoflurane vaporized at a concentration of 1.5% during the experiment (Albert-Weißenberger et al. 2012; El-Gazar et al. 2019). Isoflurane was selected for its rapid recovery profile; however, since it has been reported to modulate neuroinflammatory responses (Statler et al. 2006; Tawfeeq et al. 2009) and have neuroprotective potentials its use was standardized across all groups to eliminate confounding effects (Albert-Weißenberger et al. 2012; El-Gazar et al. 2024b; Selim et al. 2025a). Only anesthetized animals in the RTBI and RTBI + ALO received one hit per day for five consecutive days. The use of five repeated hits was intended to simulate real-life human scenarios, where individuals may experience multiple head traumas within days or weeks before complete recovery from prior injury (Mouzon et al. 2014). The 24-h gap between hits was selected because the rat brain is most vulnerable during that time (Prins et al. 2013; Grant et al. 2018).

### Behavioral Assessment

Behavioral assessments were performed on the 6th and 7th days of starting treatment.

#### Open Field Test

The open-field test is used to evaluate general locomotor activity, exploratory behavior, and anxiety-related behavior in rodents. In TBI models, it provides insight into the impact of brain injury on spontaneous movement and anxiety level (Bansal and Deshmukh 2018). Using a square box (100 × 100 × 40 cm) made of wood with a polished black floor divided into 16 equal squares by white lines (Gendy et al. 2023), the spontaneous locomotor activity was assessed. Each rat was individually placed in the center of the apparatus and given 10 min to explore. Rearing and grooming frequency, distance traveled, mean speed, line crossing, and immobility time were all recorded using an overhead camera. Following the testing of each rat, the floor and walls were thoroughly cleaned and dried with 70% ethanol to reduce scent cue bias. Behavioral assessments of animals were performed using ANY-maze video tracking software (version 7.36, Wood Dale, USA).

#### Forced Swimming Test

The forced swimming test is primarily used to evaluate depressive-like behavior and despair in rodent models, including those with TBI; increased immobility time in FST after TBI indicates depression-like behavior, reflecting affective consequences of brain injury (Bansal and Deshmukh 2018). A cylindrical tank (40 cm height × 22 cm diameter) filled with tap water thermostatically controlled to be 25 °C to the 25 cm mark (Arab et al. 2023) was used, where rats cannot support themselves by using their feet to touch the ground. The test was applied for 5 min. At the end of the test, the rats were removed from the cylinders, left to dry, and returned to their home cages. The water in the cylinders was changed after every other trial to avoid confounding results from urine or feces. The test was videotaped for later scoring for the following parameters:

- Immobility time- The amount of time, measured in seconds, that the animal remained floating passively in the water in an upright position.
- Climbing time- time in seconds during which animals vigorously moved to force out from the side of the tank wall.

### Tissue Preparation

Following the behavioral tests, animals were sacrificed under deep anesthesia with thiopental sodium (50 mg/kg) and decapitated. Three whole brains from each group were collected and preserved in 10% neutral buffered formalin for histological analysis. For the remaining animals in each group (*n* = 6), the right cortex was isolated, and the collected tissues were divided into two parts. One part was homogenized in phosphate-buffered saline (pH = 7) for ELISA, whereas the other was treated with RNAlater^®^ solution for a quantitative real-time polymerase chain reaction (qRT-PCR) to assess relative gene expression of miRNAs-322 and 125b.

### Histopathological Investigation

Whole brains preserved in 10% neutral buffered formalin were routinely processed using alcohol and xylene and then embedded in molten paraffin wax. Longitudinal sagittal Sections of five-micrometer thick were prepared and stained with hematoxylin and eosin (H&E) for light microscopic examination of the right cerebral cortex (Bancroft and Gamble 2008).

### Nissl Staining and Determination of Nissl Positive Neurons

Sections of five-micrometer thickness were cut and stained with Nissl’s stain for evaluation of neuronal degeneration. Five random non-overlapping microscopic fields were evaluated from each group by counting normal light-stained neurons in relation to the total number of neurons to detect the Nissl positive neurons in each group (El-Gazar et al. 2024a).

### Immunohistochemistry

For immune staining, 5-µm thick tissue sections were prepared on adhesive slides, rehydrated, and subjected to heat-induced epitope retrieval. Afterward, the tissue sections were incubated with primary antibodies including, anti-NF-қB (Santa Cruz Biotechnology, Inc; Cat. No sc-8008), anti-TNF-α (Santa Cruz Biotechnology, Inc; Cat. No sc-52746) and anti-HO-1 (Santa Cruz Biotechnology, Inc; Cat. No sc-390991) at a dilution of 1:200 and anti-Nrf2 (Proteintech, Germany; Cat. No 16396-1-AP) at a dilution of 1:300 for an hour at room temperature. After washing, the ready to use anti-rabbit/mouse HRP-labelled detection kit (Cat. No.BSB-0001, BioSB, USA) was used in compliance with the manufacturer’s instructions to develop the reaction. Negative control slides were obtained by escaping incubation with primary antibodies. Positive immune expression was quantified by calculating the No. of positive cells of brown-stained cells in five random non-overlapping microscopic fields in each group utilizing ImageJ Software version 1.46a (National Institute of Health, MD, USA).

### ELISA Parameters

ELISA kits were used for the measurement of the homogenate contents of Aβ1–42 (Elabscience, Tx, USA; Cat. No E-EL-R1486), Tau (MyBioSource, Ca, USA; Cat. No MBS741214), BDNF (BT LAB, SH, CH; Cat. No E0476Ra), TrkB (Biosensis, Thebarton, SA; Cat. No BEK-2178-1P), GRP78 (LSBio, WA, USA; Cat. No LS-F32597), and ATF6 (SunLong, HZ, CH; Cat. No SL1662Ra) according to the manufacturers’ instructions.

### Determination of Cortical MiRNA-322 and miRNA-125b Gene Expression

The cortical miRNA-322 and miRNA-125b gene expression levels were determined using qRT-PCR. Total RNA was extracted from the right cortex homogenates using the RNeasy Mini Kit (Cat. # 74104). Using the Quantitect SYBR^®^ Green PCR kit (Cat. # 204141) and methodology for mature miRNAs quantitative evaluation and the miR-322 and miR-125b primers, quantitative RT-PCR was carried out in a total volume of 25 µL per reaction volume. The cycling conditions for SYBR green real time PCR are fully presented in supplementary data. The primer sequences of miR-322, miR-125b′ and U6 (housekeeping gene) are listed in Table 1 Amplification curves and Ct values were determined by the Stratagene MX3005P software. To estimate the variation of gene expression on the RNA of the different samples, the CT of each sample was compared with that of the control group according to the 2^− ΔΔct^ method stated by Yuan et al. (2006).

**Table 1:** The primers sequences

Gene	Primer sequence (5’−3’)	Reference
MiRNA-322	CTCGCTGACTCCGAAGGGA	Designed in the current study using Primer-BLAST (https://www.ncbi.nlm.nih.gov/tools/primer-blast/index.cgi?LINK_LOC=Blast Home).
	CAGCGCTTCATGTTTTGAACC	
miRNA-125b	cccccGCTAGCTCTTGTTTTGCTTTGCTTTGTC	(Chen et al. 2019)
	cccGAATTCACCAAATTTCCAGGATGCAA	
U6 (housekeeping)	GCTTCGGCAGCACATATACTAAAAT	(Chen et al. 2013)
	CGCTTCACGAATTTGCGTGTCAT	

### Statistical Analysis

The normality of quantitative data was assessed using Shapiro-Wilk normality test. Then, parametric data were analyzed using one-way analysis of variance (ANOVA) followed by Tukey’s post-hoc test for multiple comparisons. Statistical significance was set at a p-value of less than 0.05. All results were presented as mean values with their corresponding standard deviation (SD). Statistical analysis and figure preparation were performed using GraphPad Prism software, version 5.01 (San Diego, California, USA). The descriptive statistics (including the mean, standard deviation (SD), and standard error of the mean (SEM)) are available in Supplementary File (1).

## Results

### Effect of ALO on Behavioral Activity after RTBI Induction Using Open Field Test

**Fig. 1:**
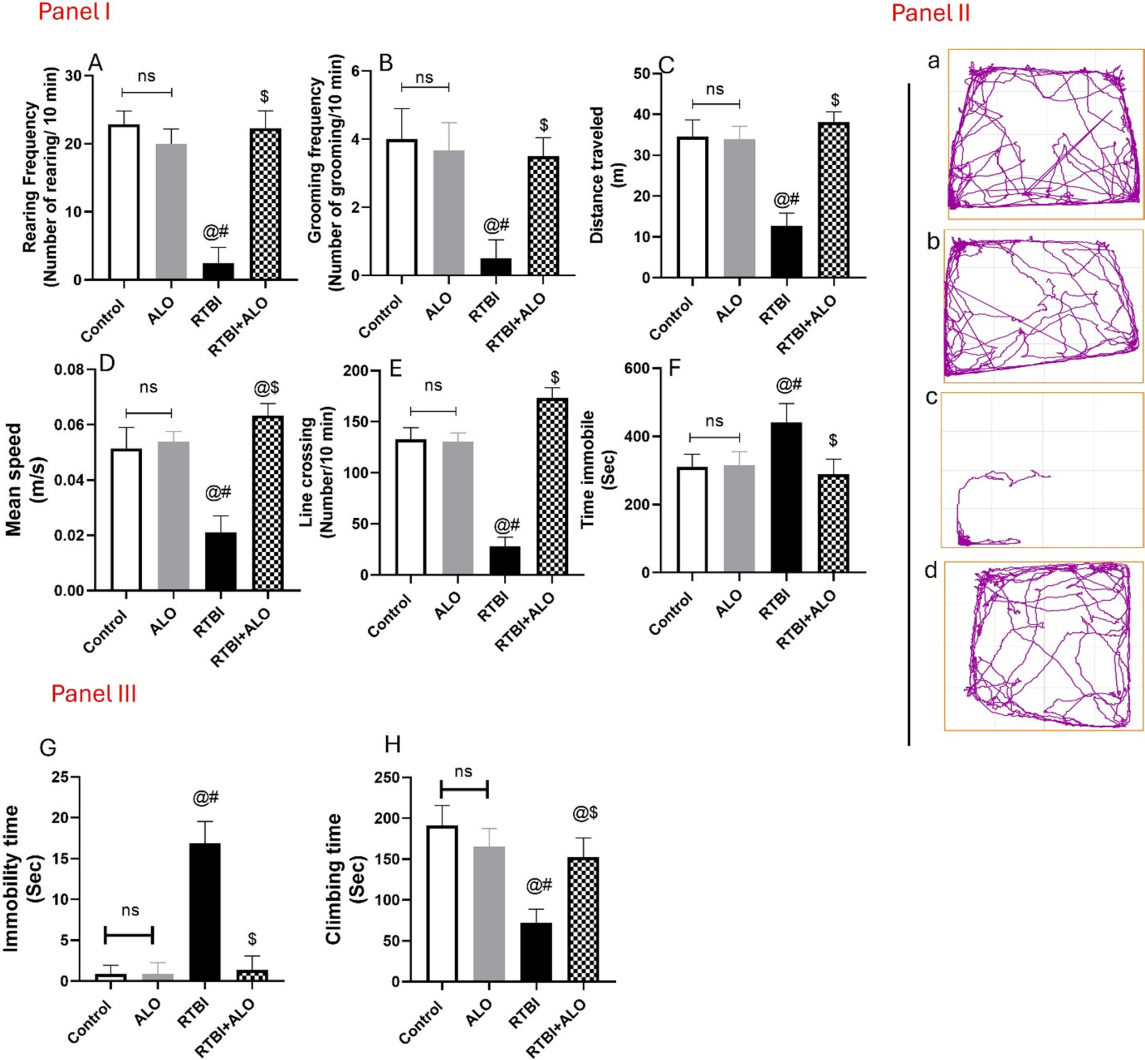
Impact of ALO on locomotor activity and behavioral changes after RTBI induction using the open field and forced swimming tests. Panel (**I**) depicts (**A**) the rearing frequency, (**B**) grooming frequency, (**C**) distance traveled, (**D**) mean speed, (**E**) line crossing, and (**F**) time immobile. Panel (**II**) displays images of (**a**) control, (**b**) ALO, (**c**) RTBI, and (**d**) RTBI + ALO that were obtained from ANY-Maze video monitoring software (Stoelting Co., USA). Panel (**III**) Impact of ALO on (**G**) immobility time and (H) climbing time using the forced swimming test after RTBI induction. Data are displayed as mean ± SD (*n* = *9*) and were analyzed by one-way ANOVA followed by Tukey’s test. The differences were considered significant at *P* < 0.05 as compared to (@) the control, (#) ALO, and ($) RTBI groups. ALO: alogliptin; ns: non-significant; RTBI: repetitive traumatic brain injury; RTBI + ALO: repetitive traumatic brain injury post-treated with alogliptin orally for seven days

Rats exposed to RTBI showed a significant decrease in exploration and locomotor activity as evidenced by the reduction in rearing frequency, grooming frequency, distance traveled, mean speed, and line crossing by 89.3%, 88.9%, 64.2%, 59%, and 81%, respectively, and a 1.4-fold increase in the time being immobile as compared to the records of the control group (*P* < 0.0001). However, ALO post-treatment following RTBI induction increased the rearing frequency (F (3,32) = 202), grooming frequency (F (3,32) = 46.32), distance traveled (F (3,32) = 115.8), mean speed (F (3,32) = 97.3), and line crossing (F (3,32) = 352.7) by 9.23, 8.25, 3.06, 3, and 6.78-fold respectively, and reduced the time being immobile (F (3,32) = 19.37) by 32.5%, displaying an enhanced behavioral record, as gathered in Fig. 1.

### Effect of ALO on Rats’ Behavioral Activity after RTBI Induction Using the Forced Swimming Test

RTBI significantly increased the immobility time by 19-fold when compared to that of the control group. On the other hand, the ALO post-treated group displayed a markedly decreased immobility time by 92.3% when compared to that of the RTBI group (F (3,32) = 173, *P* < 0.0001) (Fig. 1G). Furthermore, rats exposed to repetitive trauma presented a marked reduction in climbing time to reach 38% of the control value. Nevertheless, ALO post-treatment after repeated trauma induction elevated the climbing time by 2.1-fold relevant to that of the RTBI untreated group (F (3,32) = 48.93, *P* < 0.0001) (Fig. 1H).

### Effect of ALO on Brain Morphology and Cerebral Cortex Histoarchitecture after RTBI Induction

**Fig. 2:**
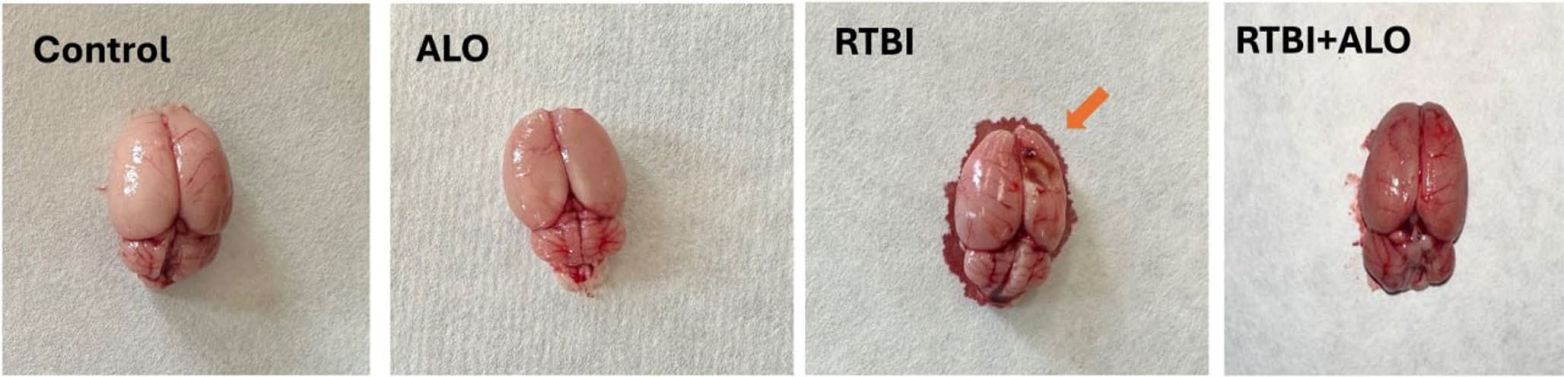
Impact of ALO on brain morphological appearance after RTBI induction. ALO: alogliptin; RTBI: repetitive traumatic brain injury; RTBI + ALO: repetitive traumatic brain injury post-treated with alogliptin orally for seven days; orange arrow: impact of five repetitive hits induced by weight drop model on the right interior frontal area

**Fig. 3:**
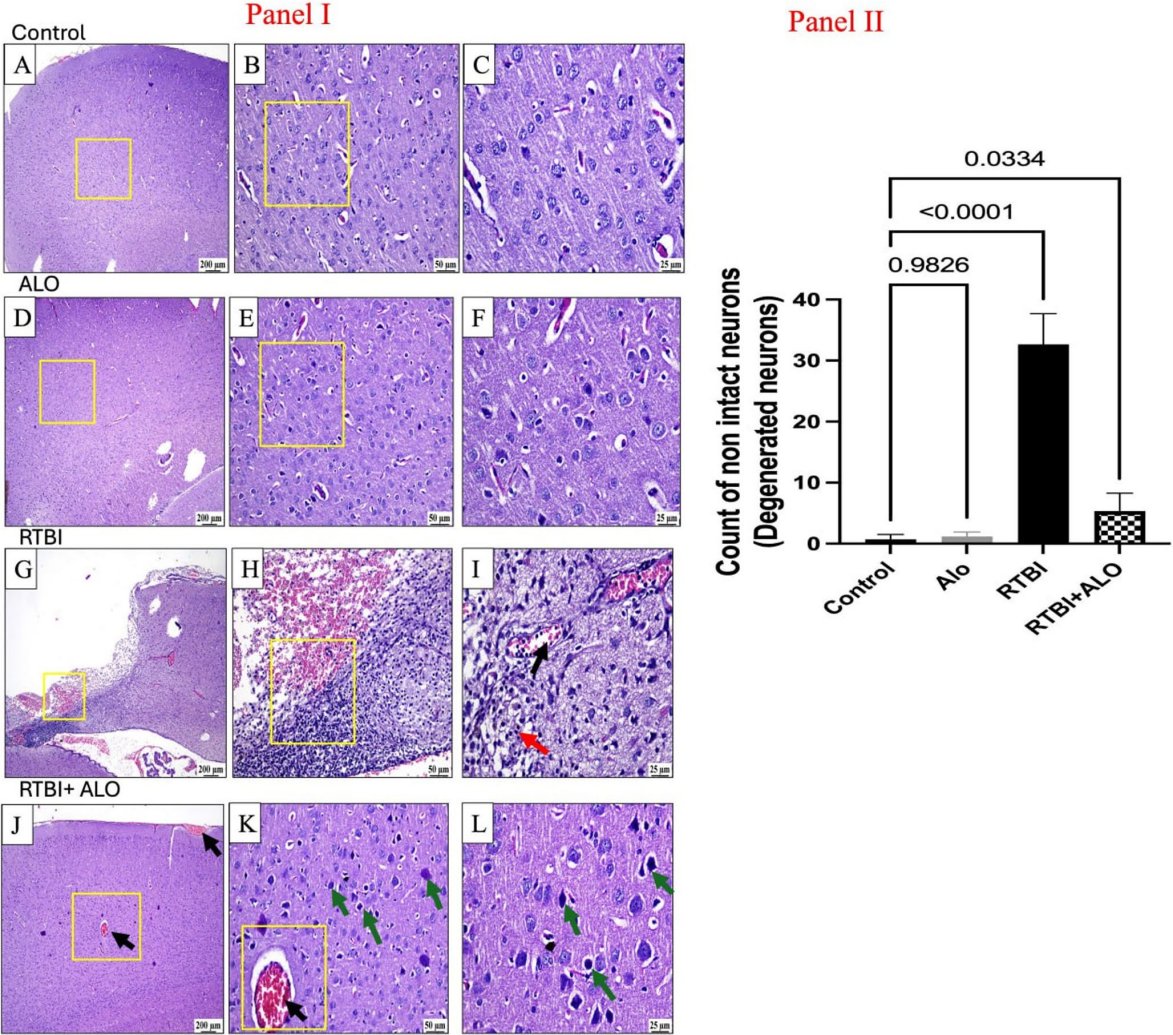
Representative photomicrographs of **H**&**E**-stained cerebral cortex of rats. Panel (**I**) the magnified regions (yellow square), control (**A**, **B**, and **C**) & alogliptin (**D**, **E**, and **F**) groups show normal cerebral cortex. The RTBI (**G**, **H**, and **I**) group shows marked loss in the cerebral cortex, hemorrhage, intense glial infiltration, distinct blood vessels (black arrow), malacia, and gliosis (red arrow). The RTBI + alogliptin (**J**, **K **& **L**) group shows congested blood vessels (black arrow) and some dark degenerating neurons (green arrow). Panel (II) count of degenerated neurons. ALO: alogliptin; RTBI: repetitive traumatic brain injury; RTBI + ALO: repetitive traumatic brain injury post-treated with alogliptin orally for seven days

The captured photos of the whole brain and photomicrographs of control rats and naive rats treated with alogliptin showed an apparently normal morphology and cerebral cortex. Nevertheless, the captured photos of the whole brains of the RTBI group showed the impact of five repetitive hits induced by the weight drop model on the right interior frontal area (orange arrow) as demonstrated in Figs. 2 and 3. Figure 3 demonstrates the microscopic examination of the cerebral cortical sections, which revealed marked cerebral damage with multiple areas of malacia and gliosis (red arrow) and with intense hemorrhage in the examined sections as compared to normal brain morphology and cerebral cortex histological findings of the control group. In contrast, treatment with alogliptin for seven days after RTBI preserved the brain morphology and cerebral cortex histoarchitecture with the existence of some dilated blood vessels and some dark degenerating neurons (green arrow). Panel II supports these observations, showing a significant increase in the count of degenerated neurons in the RTBI group compared with the control and alogliptin-only groups (*p* < 0.0001 and *p* = 0.0334, respectively), whereas alogliptin treatment after RTBI markedly reduced neuronal degeneration (*p* < 0.0001 vs. RTBI).

### Effect of ALO on Neuronal Degeneration after RTBI Induction

**Fig. 4:**
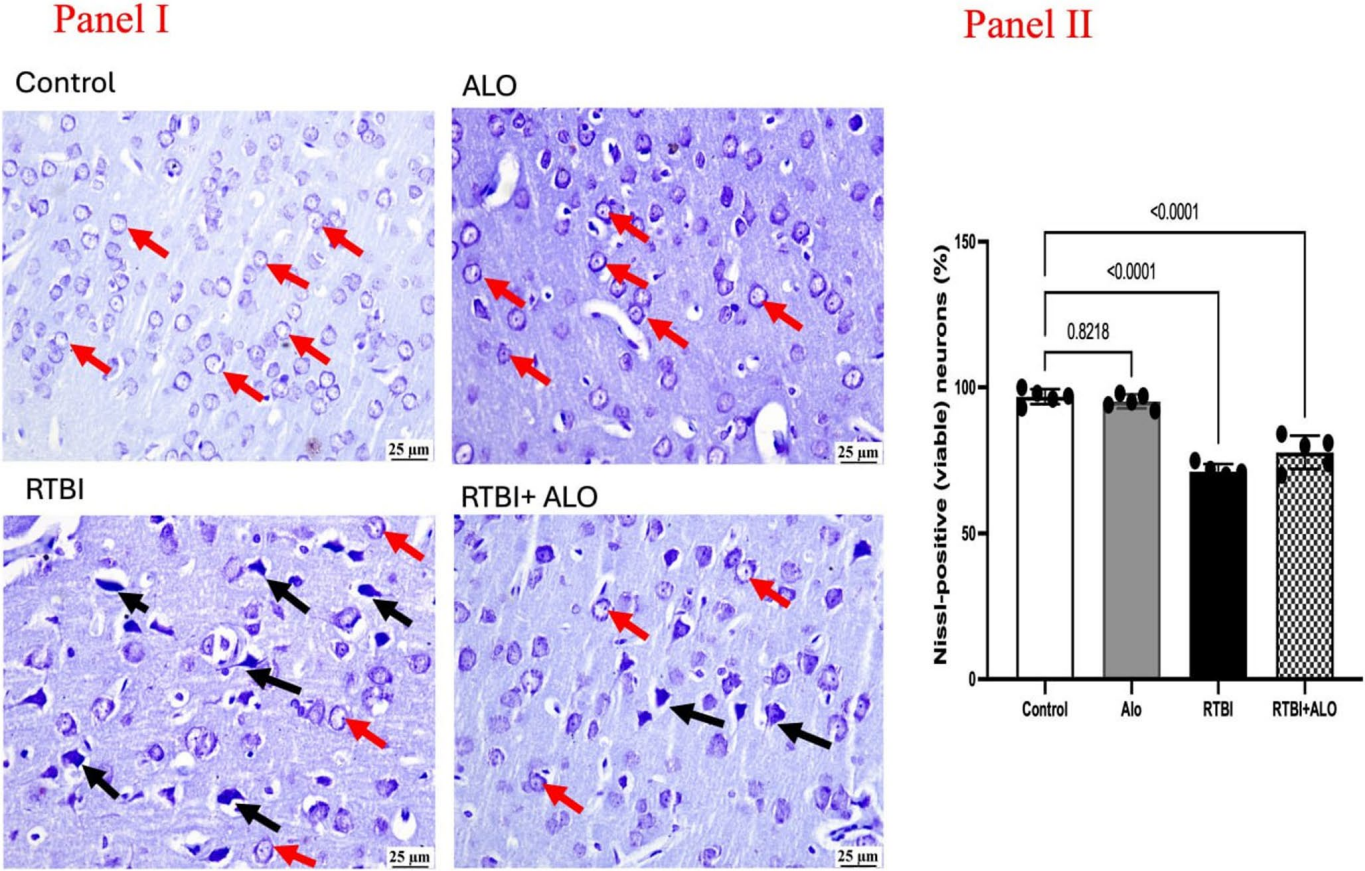
Impact of ALO post-treatment on neuronal degeneration using Nissl stain after RTBI. The cerebral cortices of both the control and ALO groups exhibited normal light-stained neurons. However, the cerebral cortex of the RTBI group showed numerous dark degenerating neurons (black arrow), Nissl viable positive neurons (red arrow), while the RTBI + ALO group showed fewer degenerating cells (black arrow). The differences were considered statistically significant at *P* < 0.05 as compared to the (@) Control, (#) ALO, and ($) RTBI groups. ALO: alogliptin; ns: non-significant; RTBI: repetitive traumatic brain injury; RTBI + ALO: repetitive traumatic brain injury post-treated with alogliptin orally for seven days

The neuronal degeneration in the different experimental groups was assessed using Nissl staining (Fig. 4). The cerebral cortices of both the control and ALO groups displayed normal light-stained neurons, whereas the cerebral cortex of the RTBI group showed numerous dark degenerating neurons. Notably, ALO post-treatment significantly reduced the number of degenerating neurons in comparison to the RTBI group (Panel I). A graphical presentation of Nissl positive neurons showed that rats with RTBI recorded a marked reduction in the number of healthy neurons to reach 75% as compared to the records of healthy rats. On the other side, the ALO post-treated group showed a significant rise in the estimated Nissl positive neurons by 1.1-fold when compared to the RTBI group (F (3,16) = 63.07, *P* < 0.0001) (Panel II).

### Effect of ALO on Abnormal Protein Aggregation after RTBI Induction

**Fig. 5:**
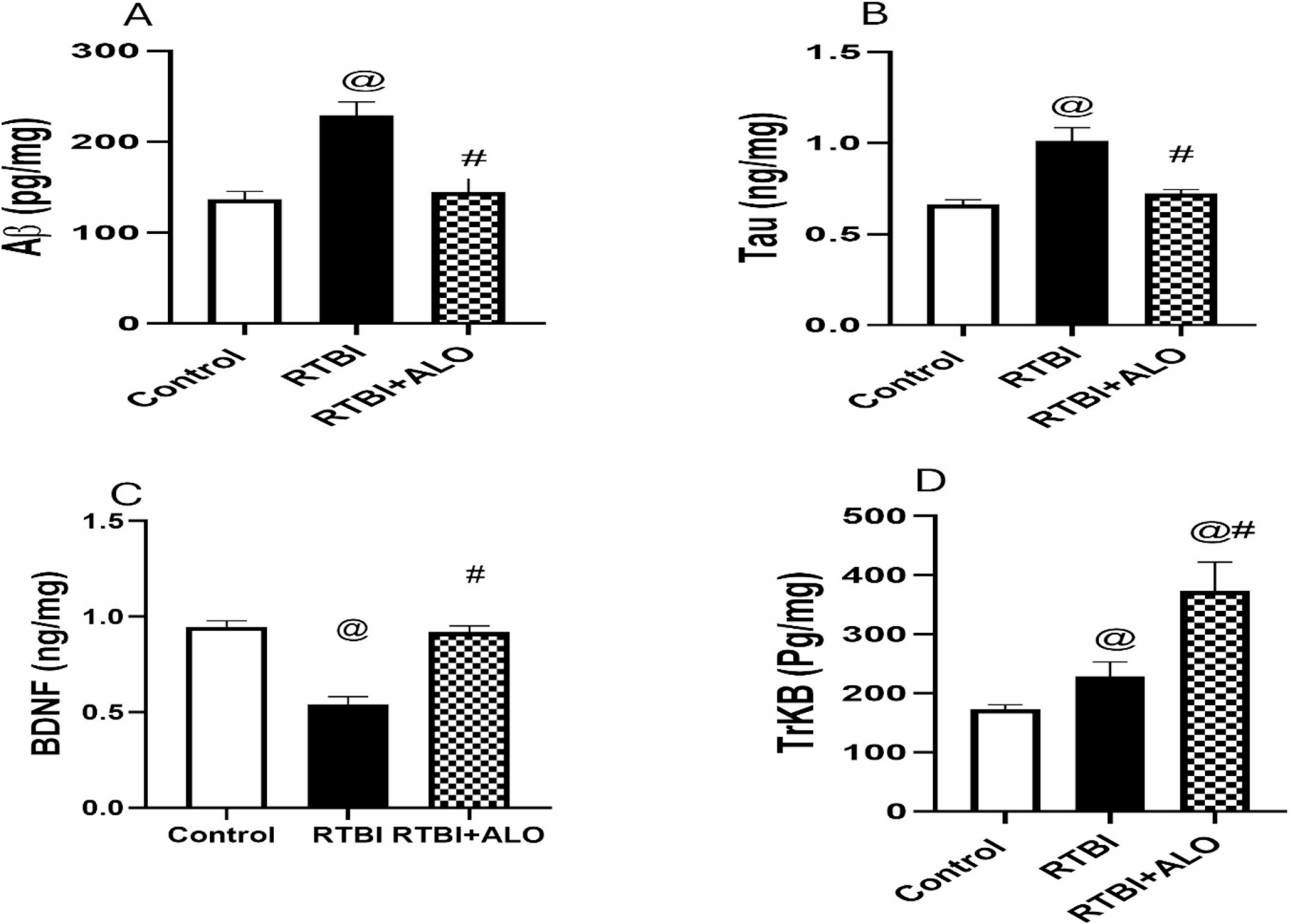
Effect of alogliptin on cortical contents of Aβ (**A**), Tau (**B**), BDNF (**C**), and TrKB (**D**) after RTBI induction in rats. Data are presented as mean ± S.D (*n* = *6*) and were analyzed using one-way ANOVA followed by Tukey’s post hoc test. The differences were considered significant at *P* < 0.05 as compared to the (@) Control and (#) RTBI groups. Aβ: β-amyloid; BDNF: brain-derived neurotrophic factor; TrKB: tropomyosin receptor kinase B; RTBI: repetitive traumatic brain injury; RTBI + ALO: repetitive traumatic brain injury post-treated with alogliptin orally for seven days

RTBI obviously elevated the cortical contents of Aβ (1.7-fold; F (2,15) = 78.48) and Tau (1.5-fold; F (2,15) = 101.2) in comparison to the control group (*P* < 0.0001). On the other hand, oral administration of alogliptin (20 mg/kg) resulted in a marked decline in cortical contents of Aβ and Tau by 37% and 28.6%, respectively, compared to those exposed only to RTBI insult (Fig. 5).

### Effect of ALO on Cortical Contents of BDNF and TrKB after RTBI Induction

In comparison with the control group, the RTBI group showed a significant decrease in BDNF by 42.8% and an increase in TrKB (1.3-fold) cortical contents. On the other hand, the ALO post-treated group exhibited a marked increase in cortical contents of both (C) BDNF (1.7-fold; F (2,15) = 259.2) and (D) TrKB (1.6-fold; F (2,15) = 62.84) relevant to the RTBI group at *P* < 0.0001 (Fig. 5).

### Effect of ALO on Nrf2 and HO-1 Expression after RTBI Induction

**Fig. 6:**
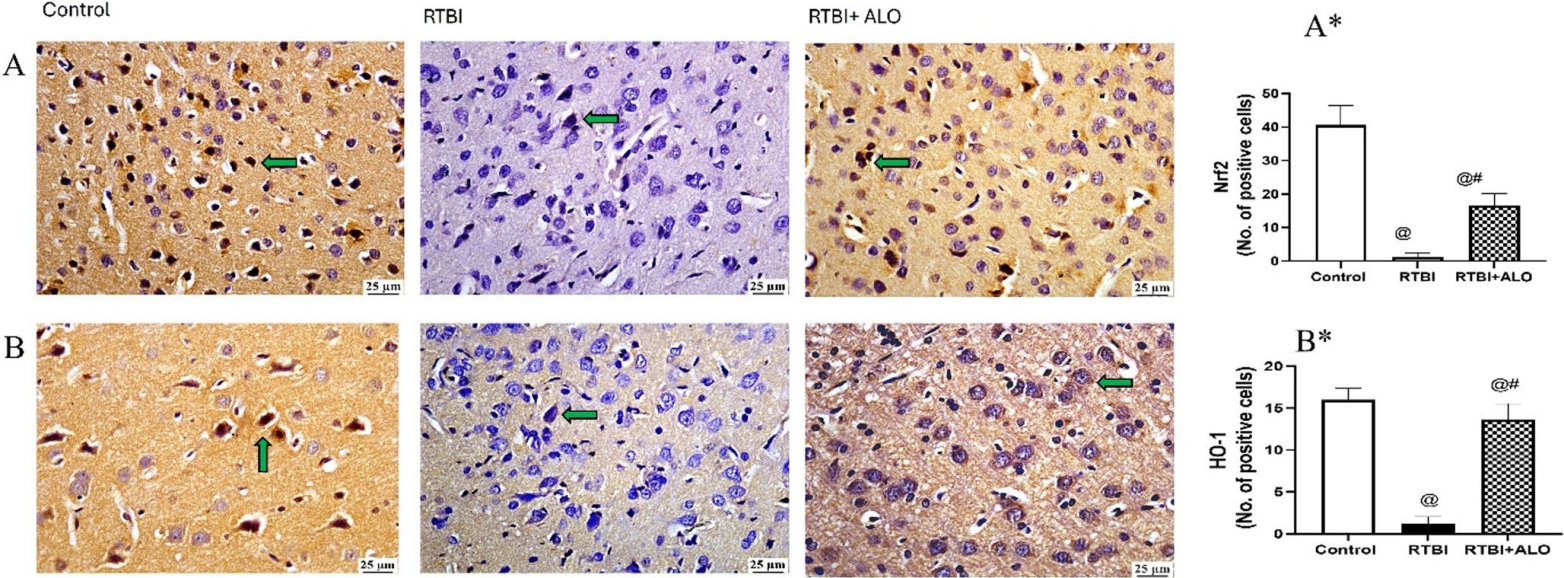
ALO treatment increased cerebral cortex immunohistochemical expression of Nrf2 (**A**&A*) and HO-1 (**B**&B*) after RTBI induction in rats. Panels (A&B) illustrate the pronounced brown positive staining of Nrf2 and HO-1, respectively, in the control. However, the RTBI-exposed group displayed an absence of Nrf2 and HO-1 expression, as indicated by blue staining. In contrast, the ALO-treated group after RTBI exhibited a notable increase of Nrf2 and HO-1 positive nuclei in comparison to the RTBI group. Panel (A*) represents No. of positive cells of Nrf2 articulated as the mean of 5 non-overlapping microscopic fields (*n* = 3/group) ± SD, while (B*) represents No. of positive cells of HO-1 articulated as the mean of 5 non-overlapping microscopic fields (*n* = 3/group) ± SD. The differences were considered statistically significant at *P* < 0.05 as compared to the (@) Control and (#) RTBI groups. Green arrow: Positive cells; Nrf2: nuclear factor erythroid 2-related factor 2; HO-1: heme oxygenase-1; RTBI: repetitive traumatic brain injury; RTBI + ALO: repetitive traumatic brain injury post treated with alogliptin orally for seven days

RTBI markedly diminished the cortical immuno-expression of the antioxidant Nrf2 (A&A*) and its downstream molecule HO-1 (B&B*) (Fig. 6) by 86.7% (F (2,12) = 445.2) and 72.8% (F (2,12) = 245), respectively, as compared to the control group. ALO post-treatment showed antioxidant capacity by increasing Nrf2 (6-fold) and HO-1 (2.5-fold) immuno-expression in comparison to the RTBI untreated group at *P* < 0.0001.

### Effect of ALO on the Levels of TNF-α and NF-κB after RTBI Induction

**Fig. 7:**
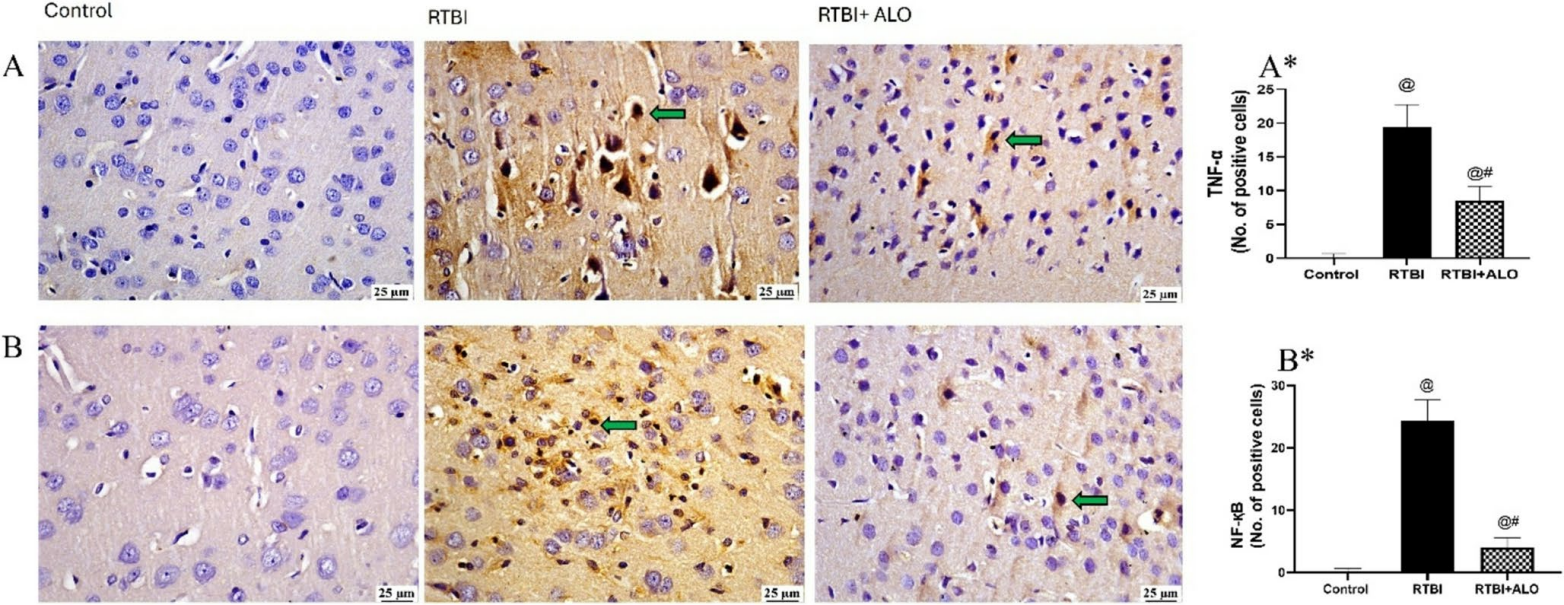
ALO post-treatment reduced the cerebral cortex immunohistochemical expression of TNF-α (**A**&A*) and NF-қB (**B**&B*) after RTBI induction in rats. Panels (**A **& **B**) illustrate pronounced brown positive staining of TNF-α and NF-қB, respectively, in the RTBI-exposed group relative to the absence of expression in the control group, whereas the ALO-treated group after RTBI exhibits a notable decrease of TNF-α and NF-қB positive nuclei, respectively. Panel (A*) represents No. of positive cells of TNF-α articulated as the mean of 5 non-overlapping microscopic fields (n = 3/group) ± SD, whereas (B*) represents No. of positive cells of NF-қB articulated as the mean of 5 non-overlapping microscopic fields (n = 3/group) ± SD. The differences were considered statistically significant at *P* < 0.05 as compared to the (@) Control and (#) RTBI groups. Green arrow: Positive cells; TNF-α: tumor necrosis factor alpha; NF-қB: nuclear factor-kappa B; RTBI: repetitive traumatic brain injury; RTBI + ALO: repetitive traumatic brain injury post treated with alogliptin orally for seven days

A massive increase in the inflammatory process in the RTBI group, indicated by a significant increase in the immuno-expression of TNF-α (A&A*) and NF-қB (B&B*) (Fig. 7) (9.2- and 15.7-fold, respectively) when compared to the control group. In comparison with the RTBI group, the ALO post-treated group showed a marked reduction by 40.7% (F (2,12) = 92.8) and 52.3% (F (2,12) = 128) in the immuno-expression of TNF-α and NF-қB, respectively, at *P* < 0.0001.

### Effect of ALO on miRNA Gene Expression

**Fig. 8:**
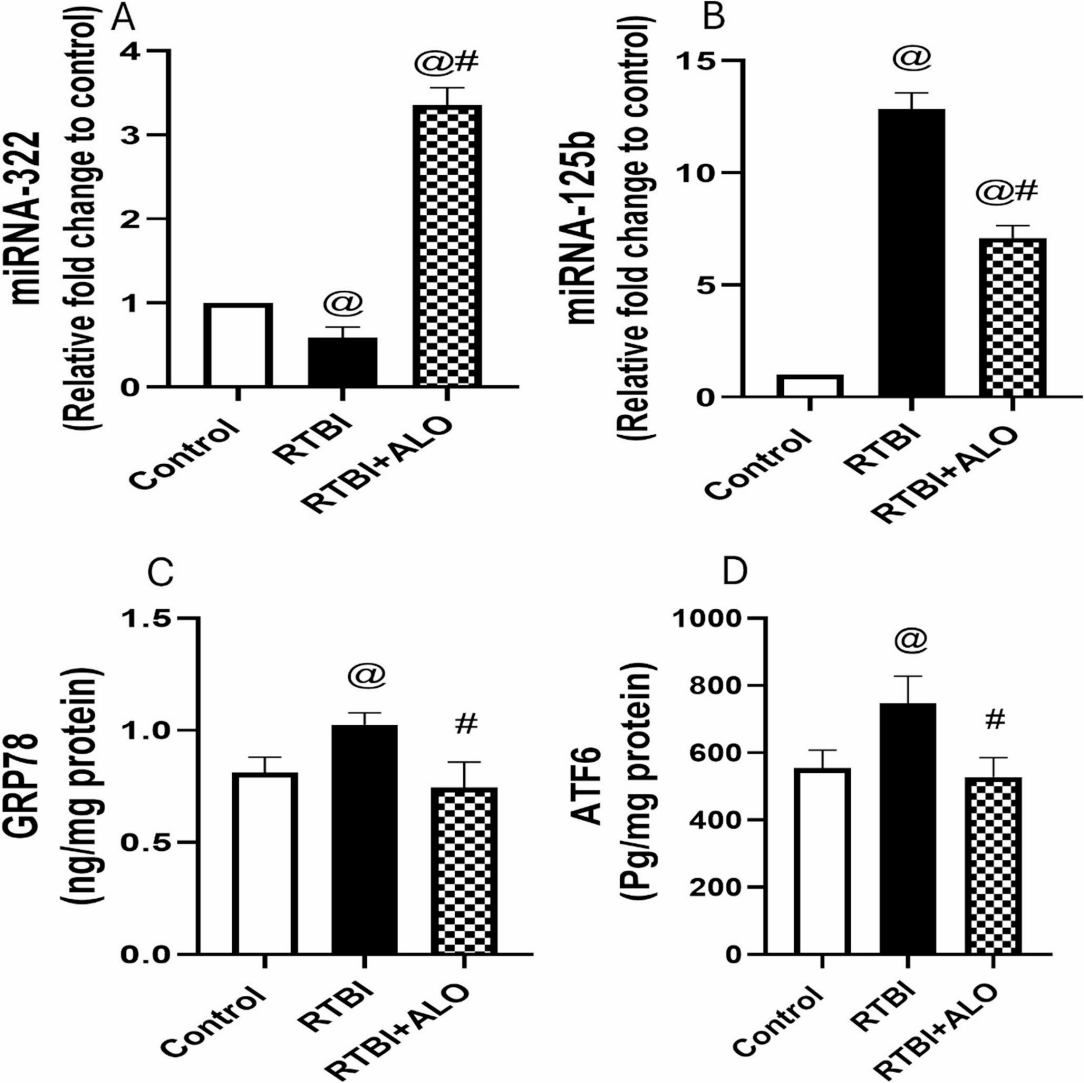
Changes in right cortical gene expression of miRNA-322 (**A**) and miRNA-125b (**B**) and impact of ALO treatment on the cortical contents of GRP78 (**C**) and ATF6 (**D**) after RTBI induction in rats. The statistical analysis was conducted using one-way ANOVA and Tukey’s post hoc multiple comparisons test. The values are expressed as mean ± SD (n = 5). The differences were considered statistically significant at P < 0.05 as compared to the (@) Control and (#) RTBI groups. GRP78: glucose-regulated protein 78; ATF6: activating transcription factor 6; RTBI: repetitive traumatic brain injury; RTBI + ALO: repetitive traumatic brain injury post-treated with alogliptin orally for seven days

Untreated RTBI rats exhibited a significant decrease in gene expression of miRNA-322 (41.39%; F (2,12) = 574.1) and a significant increase in gene expression of miRNA-125b (12.8-fold; F (2,12) = 636.8) in comparison to healthy rats (Fig. 8). However, ALO administration effectively increased the gene expression of miRNA-322 (5.7-fold) and mitigated the alteration in miRNA-125b by 44.7% when compared to the RTBI group at *P* < 0.0001.

### Effect of ALO on ER Stress after RTBI Induction in Rats

GRP78 and ATF6 cortical contents were significantly increased (1.3- and 1.4-fold, respectively) in the RTBI group when compared to the control group as depicted in Fig. 8. However, the ALO post-treated group exhibited significant reduction by 27% and 29.4% in GRP78 and ATF6 levels, respectively, in comparison to the RTBI group (F (2,12) = 15.68; F (2,12) = 16.92 at *P* < 0.0004 and *P* < 0.0003, respectively).

## Discussion

The primary objective of this study is to investigate the neuroprotective effect of alogliptin (ALO) on endoplasmic reticulum (ER) stress induced by repetitive traumatic brain injury (RTBI) in a rat model. The acquired results suggest that ALO, an antidiabetic drug, could be a promising candidate for preventing RTBI-induced secondary impacts and neurodegeneration. ALO increased locomotor activity, reduced depressive behavior and cognitive dysfunction, and increased cortical contents of BDNF and TrKB, promoting neuronal protection and plasticity, as evidenced by restoring cortical histoarchitecture and decreasing the number of degenerated neurons. Furthermore, ALO exhibited antioxidant and anti-inflammatory potentials by modulating the cortical immune-expression of Nrf2/Ho-1 and reducing TNF-α and NF-қB immuno-expression in the cortex. Ultimately, the post-transcriptional modulation of miRNA (322&125b) by ALO increased its neuroprotective capacity. All these potentials played a role in mitigating ER stress triggered by RTBI.

In this study, RTBI rats presented a considerable reduction in locomotor activity and augmented depressive behavior, as confirmed by the open field and forced swimming test. These behavioral disturbances were documented in our previous studies (El-Gazar et al. 2024a, b), which demonstrate that RTBI destroys the locomotor function and promotes depressive behavior. The secondary injury associated with TBI may disrupt brain homeostasis, leading to elevated dipeptidyl peptidase-4 (DPP-4) activity, which has been linked to chronic neurodegenerative changes (You et al., 2023; Jiang et al. 2024). According to studies, DPP-4 may cause neuronal damage and impair healing by triggering the inflammatory response after brain trauma. Moreover, neurodegenerative disorders and cognitive impairment have been associated with elevated DPP-4 levels. (Ma et al., 2020; Razavi et al., 2024). Hence, ALO as an inhibitor DPP-4 inhibitor would be a good candidate in neurodegenerative disorders associated with high DPP-4 activity. The administration of ALO (20 mg/kg) relieved the motor abnormalities and enhanced motor coordination in RTBI-exposed rats, as shown by the open field test. ALO also decreased immobility and increased climbing time as denoted by the forced swimming test. This comes in line with previous studies, which reported that ALO treatment enhanced cognitive and motor functions in animal models of depression (Mori et al. 2017), Alzheimer’s disease (AD) (Rahman et al. 2020), and parkinsonism (Safar et al. 2021). Furthermore, RTBI caused marked histopathological changes in the cerebral cortex. These findings are in harmony with our previous report of RTBI (El-Gazar et al. 2024a). ALO administration recovered cerebral cortical architecture in rats exposed to RTBI. Neuroprotective effects of ALO have been well-documented in different experimental models (Mori et al. 2017; Safar et al. 2021; Alsemeh and Abdullah 2022; Mohammed 2023), supporting our histological findings.

RTBI is known to induce diffuse neuropathological changes involving interconnected and functionally vulnerable regions such as the hippocampus and striatum. Therefore, it is reasonable to expect that the cortical protection exerted by alogliptin in the current study may also mitigate secondary injury in these distant regions through attenuation of oxidative stress, neuroinflammatory cascades. This concept is supported by our previous work (El-Gazar et al., 2024a), where modulation of key molecular pathways in the cortex was reflected by parallel neuroprotection in the hippocampus and striatum. Furthermore, (Rahman et al. 2020) demonstrated that alogliptin exerted direct hippocampal protection by reversing insulin resistance and reducing Aβ deposition, oxidative stress, and neuroinflammation in an Aβ(1–42)-induced hippocampal injury model. In addition, DPP-4 inhibition has been shown to reduce striatal microglial ramification and neuroinflammation following cortical impact injury, suggesting a broader region-dependent neuroprotective potential beyond the site of primary trauma (Hung et al. 2020).

The alteration in behavioral activity could be a reflection of altered biological parameters as shown in this study, where RTBI caused abnormal aggregation of proteins such as Aβ and Tau, as previously reported (McKee et al. 2009). Abnormal Aβ aggregation leads to over phosphorylation of tau and tangle formation that affects neuronal function and plasticity and causes long-term behavioral disturbances (Schmechel et al. 1993). Interestingly, ALO significantly decreased the cortical contents of Aβ and Tau. These results are supported by a previous study that confirmed the ability of ALO to decrease the abnormal phosphorylation of tau and reduce neurofilament levels in an animal model of AD (Jiang et al. 2024).

Furthermore, RTBI-exposed rats presented a substantial decline in BDNF cortical content, which is essential in neuronal differentiation, survival, and synaptic plasticity (Wurzelmann et al. 2017). Although there was a decline in cortical contents of BDNF herein, there was upregulation in TrkB, the receptor for BDNF, leading to ineffective signaling and neuronal dysfunction, as the prime function of TrkB is to mediate the effects of BDNF, where BDNF selectively binds to TrkB, mediating different beneficial cellular effects (Benarroch 2015). Thus, a reasonable explanation is that cellular compensation upregulated TrKB levels to face the RTBI-mediated neurodegenerative effect. Auspiciously, ALO increased both BDNF and TrKB cortical contents, resulting in effective downstream signaling and promoting neuronal survival and plasticity (Saarelainen et al. 2003; Rantamäki et al. 2007), as evidenced herein by behavioral and histological findings. ALO is reported to markedly elevate BDNF mRNA expression in the brain. This upregulation is correlated with enhanced cognitive performance as demonstrated by improved outcomes in behavioral assessment in animal models (Mori et al. 2017). Improved behavioral performance in the open-field and forced swimming tests was correlated with higher expression of BDNF and TrkB, indicating a functional link between behavioral recovery and alogliptin-mediated neurotrophic support. These results are consistent with earlier studies showing that GLP-1 based treatments and DPP-4 inhibitors increase BDNF expression and improve synaptic function to produce neuroprotective and pro-plasticity effects (Darsalia et al. 2013; Dong et al. 2019).

RTBI leads to an imbalance between the production of reactive oxygen species (ROS) and the ability of the body to neutralize them with antioxidant systems (Ismail et al. 2020). An oxidative stress sensor, Nrf2, and its downstream detoxification enzyme, HO-1, were chosen to assess the antioxidant effect of ALO in the current work. A significant reduction in the immunohistochemical cortical expression of Nrf2 and HO-1 was observed in RTBI-exposed rats. This milieu was confirmed in our recent study of RTBI (El-Gazar et al. 2024b).

Additionally, the reduction in the antioxidant system marked herein resulted in increased inflammatory responses, which was confirmed by an elevation in the immuno-expression of TNF-α and NF-қB, two critical components involved in immune response and neuroinflammation. Numerous transcription factors can be activated by oxidative stress, resulting in the differential expression of certain genes linked to inflammatory pathways (Hussain et al. 2016), augmenting the latter results. Furthermore, post-TBI primary injury, toll-like receptors (TLRs) become activated (Demediuk et al. 1985); this activation triggers astrocytes and microglia to release reactive oxygen species (ROS), chemokines, and cytokines. These molecules contribute to inflammation and further exacerbate neuronal damage (van Noort and Bsibsi 2009). TNF-α is one of the inflammatory cytokines released as a result of ROS overproduction (Grilli et al. 1993). Elevated oxidative stress, along with increased free radical production, has been linked to TBI-induced disruption of neuronal homeostasis (Demediuk et al. 1985; Inci et al. 1998). Our study demonstrated the antioxidant and anti-inflammatory effects of ALO, which were verified herein by increased Nrf2 and HO-1 and decreased TNF-α and NF-қB cortical immune-expressions. The antioxidant and anti-inflammatory potentials of ALO were previously confirmed in different studies (Yisireyili et al. 2016; Alsemeh and Abdullah 2022; Mohammed 2023). The aforementioned modulatory effect of ALO was mirrored on histological findings, where ALO rescued the cortex and increased the Nissl positive neurons in the RTBI + ALO group. In line, oxidative stress and heightened inflammatory state damage different cellular components, contributing to neural dysfunction and finally death (Wu et al. 2022; Vitner et al. 2012).

Our study tracked, for the first time, the post-transcriptional gene modulators miRNA-322 and miRNA-125b, where RTBI significantly decreased the cortical gene expression of miRNA-322 and increased that of miRNA-125b. MiRNA-322 has been identified as an important modulator of inflammatory responses, functioning as negative regulator by targeting the NF-κB1 (p50) signaling pathway. In studies using lipopolysaccharide stimulated macrophages. MiRNA-322 suppressed the production of inflammatory cytokines, thereby promoting cell proliferation and attenuating inflammation (Zhang et al. 2017; Ruan et al. 2024). Furthermore, under conditions of ER stress miRNA-322 expression was reported to be downregulated (Groenendyk et al. 2014; Dai et al. 2015). On the other hand, miRNA-125b overexpression in primary hippocampal neurons exacerbated AD pathogenesis by increasing tau phosphorylation, which provokes neuronal death (Banzhaf-Strathmann et al. 2014; Zhang et al. 2019). Clinically, circulating miRNA-125b was documented as a potential biomarker of AD (Cogswell et al. 2008). Additionally, miRNA-125b induces inflammatory response through activation of NF-қB (Zhang et al. 2021), whereas its downregulation reduces NF-κB transcriptional targets expression (Parisi et al. 2016). Notably, suppression of miRNA-125b has been associated with improved motor neuronal survival, indicating that precise regulation of this miRNA is essential for maintaining neuronal health (Le et al. 2009; Parisi et al. 2016).

ALO reversed the effect of RTBI on the investigated miRNAs, increasing the cortical gene expression of miRNA-322 and reducing the gene expression of miRNA-125b, offering neuroprotection. To the authors’ knowledge, this is the first documentation of the effect of ALO on these two miRNAs. Formerly the upregulation of miRNA-125b gene expression was associated with a decrease in the antioxidant system (Liang et al. 2018). Moreover, the upregulation of miRNA-322 gene expression negatively regulates the inflammatory response (Zhang et al. 2017) and exhibits anti-apoptotic effects (Liu et al. 2020). These findings may explain the increase in cortical contents of Nrf2/HO-1 and the decrease in cortical immuno-expression of TNF-α and NF-қB observed in the RTBI group treated with ALO.

Oxidative stress can induce endoplasmic reticulum (ER) stress by accumulation of unfolded proteins (Ong and Logue 2023), and an exaggerated inflammatory response can disrupt ER function (Zhang et al. 2006). Moreover, miRNA-125b is significantly upregulated in ER stress (Luís et al. 2020), whereas induction of ER stress was correlated with diminished miRNA-322 abundance (Groenendyk et al. 2014), in harmony with the findings of the present study. Hence, ER stress was investigated in the current study, as it plays a substantial role in the progression of secondary brain injury after TBI (Tan et al. 2018). The aggregation of unfolded proteins after TBI may cause ER stress (Lucke-Wold et al. 2017), and ER stress also contributes to unfolded protein response (URP) (Larner et al. 2016). Besides the cognitive deficit, behavioral changes associated with TBI may be indirectly related to ER stress (Hylin et al. 2018; Ghemrawi and Khair 2020). All these facts confirm the incidence of ER stress in the current work, where rats with RTBI showed significantly increased ATF6 and GRP78 levels as compared to those of the control group. One possible interpretation is that the increase in protein misfolding enhances GRP78 dissociation from ATF6, a stress sensor, to recognize and manage the toxic protein species (Hetz 2012). On the other side, ALO-treated rats demonstrated a significant reduction in ER stress markers: ATF6 and GRP78. To the authors’ knowledge, this effect was not previously documented. A possible explanation for the modulatory effect of ALO on ER stress is its ability to increase the cortical contents of BDNF after RTBI induction. In line, a previous study showed that BDNF protects against ER stress through the downregulation of CCAAT-enhancer-binding protein homologous protein (CHOP) (Chen et al. 2007). In addition, low cortical contents of Aβ and Tau proteins observed in the RTBI + ALO group may be a consequence of enhancing the ubiquitin proteasome system (UPS) in the ER to degrade unfolded proteins, thus decreasing the toxic protein load and ER stress (Zhu et al. 2021). As previously mentioned herein, ALO treatment improved motor and cognitive functions and restored cortical structural abnormalities, which may in part be attributed to its ability to restore the folding capacity of the ER (Mori et al. 2017; Safar et al. 2021). Finally, the anti-inflammatory and antioxidant effects, in addition to upregulation of miRNA-322 and downregulation of miRNA-125b gene expression by ALO presented herein, may play a role in the modulatory effect of ALO on ER stress markers.

## Conclusion, Future directions, and Clinical Relevance

The findings of this study shed light on the multifaceted neuroprotective mechanisms of ALO against RTBI-induced secondary brain injury, highlighting its effect on ER stress and post-transcriptional modulation of miRNA (322 & 125b), as well as its antioxidant and anti-inflammatory effects. These actions were mirrored as a positive impact on behavioral and histological outcomes. Moving forward, it is imperative to investigate the long-term effects of ALO treatment post-RTBI, particularly in terms of cognitive outcomes and progression to chronic neurodegenerative states, such as chronic traumatic encephalopathy (CTE). From a translational standpoint, clinical dose optimization represents a crucial next-step assessment of sex-specific response to RTBI and ALO treatment. Growing evidence suggests that males and females may exhibit distinct pathophysiological responses to brain injury, influenced by hormonal milieu, inflammatory reactivity, and gene expression patterns. Another clinical direction is to assess the contribution of the GLP-1 signaling pathway to the observed effect of ALO to clarify whether the neuroprotective effects of ALO are mediated directly through enhanced GLP-1 signaling or alternative pathways, such as antioxidant or anti-inflammatory effects or miRNA modulation.

## Limitations of the Study

First, only male rats were used in the present study, which may limit its translational relevance. Indeed, sex difference was amply reported, and several studies found that it impacts both behavioral and biochemical assessments (McCorkle et al. 2022; Scott et al. 2022; Fox et al. 2023; Li et al. 2024) either due to hormonal difference or even genetic variation. However, the present study did not aim to explore the role of sex difference on the assessed drug or the studied signaling pathway, and accordingly, to evade the effect of another factor other than the drug, only male animals were utilized. However, once the findings have been established and confirmed, it is advisable to perform further research that aims at studying the potential impact of gender on traumatic brain injury results using both female and male rats. The second limitation is the short-term evaluation of outcomes in the current study. Future research should evaluate the long-term effects of alogliptin on traumatic brain injury with respect to both safety and efficacy. Another limitation is that a single dose of alogliptin (20 mg/kg) (Akita et al. 2015; Kabel 2018) was investigated. This dose was selected based on previous preclinical studies, where it showed significant anti-inflammatory and neuroprotective effects in rodent models of neurological injury or metabolic dysfunction (Rahman et al. 2020; Alsemeh and Abdullah 2022; Mohammed 2023; Botros et al. 2024; Selim et al. 2025b). Notably, this dose is not equivalent to the human dose of alogliptin according to the FDA body surface area conversion (Nair and Jacob 2016). However, taking into consideration that alogliptin is intended for chronic use in humans, using rigid human equivalent dose (HED) is scientifically unjustified when translating chronic human dosing to short-term rodent studies. Thus, body surface area (BSA)-based HED conversions fail to account for critical physiological and pharmacokinetic differences between species (Blanchard and Smoliga 2015). Accordingly, the used dose of alogliptin in the present study is in line with previous studies on the effects of alogliptin in rodents although not equivalent to the human dose, where short-term rodent dosing cannot replicate chronic human exposure using the same equivalent dose due to metabolic, pharmacokinetic, and physiologic differences. Future research should evaluate the PERK and IRE1 arms of ER stress. Although this study focused on the right cerebral cortex as the primary site of impact, RTBI is known to cause diffuse injury involving connected regions such as the hippocampus and striatum. This RTBI model has previously been shown to induce hippocampal degeneration (El-Gazar et al. 2024a), and future work will investigate the region-specific neuroprotective effects of alogliptin beyond the cortex.

## Supplementary Information

Below is the link to the electronic supplementary material.

Supplementary Material 1 (DOCX 5.78 MB)

## Data Availability

Data will be made available on request.
