# Supplementary material for: Repositioning of Alogliptin to Mitigate Secondary Injury Induced by Repetitive TBI: Potential Role of its Antioxidant and Anti- Inflammatory Effects
Source: J Neuroimmune Pharmacol. 2026 Jan 13;21(1):6. doi: 10.1007/s11481-025-10271-w (PMC12799724; doi:10.1007/s11481-025-10271-w)

- **The sharp-edged weight used to induce repeated traumatic brain injury (RTBI).**

**
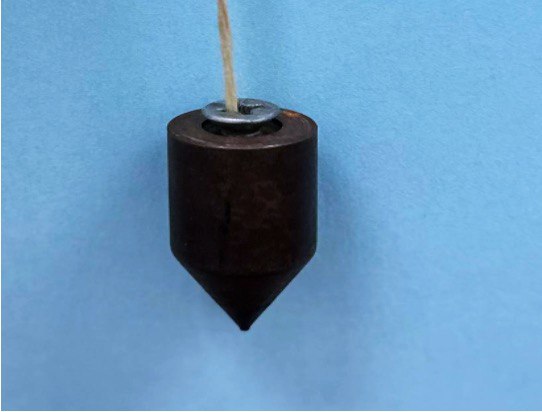
**

- **Descriptive statistics of the behavioral tests in Fig. 1**

**1-Descriptive statistics of the open-field test**

|  | Control | | | ALO | | | RTBI | | | RTBI+ALO | | |
| --- | --- | --- | --- | --- | --- | --- | --- | --- | --- | --- | --- | --- |
|  | **Mean** | **SD** | **SEM** | **Mean** | **SD** | **SEM** | **Mean** | **SD** | **SEM** | **Mean** | **SD** | **SEM** |
| Rearing  (n=9) | 22.78 | 1.716 | 0.572 | 21.22 | 2.224 | 0.7412 | 2.444^@#^ | .968 | 0.1894 | 22.56^$^ | 2.297 | 0.7658 |
| Grooming (n=9) | 4 | 0.866 | 0.2887 | 3.778 | 0.8333 | 0.2778 | 0.4444^@#^ | .127 | 0.0757 | 3.667^$^ | 0.7071 | 0.2357 |
| Distance travelled (n=9) | 34.71 | 4.017 | 1.339 | 33.35 | 3.016 | 1.005 | 12.43^@#^ | 3.099 | 1.033 | 38.09^$^ | 2.712 | 0.9041 |
| Time immobile (n=9) | 311.6 | 38.76 | 12.92 | 311.8 | 41.21 | 13.74 | 441^@#^ | 56.84 | 18.95 | 297.6^$^ | 44.7 | 14.90 |
| Line crossing (n=9) | 132.8 | 11.39 | 3.796 | 130.4 | 8.691 | 2.897 | 25.22^@#^ | 10.11 | 2.370 | 173.2^$^ | 10.05 | 3.349 |
| Mean speed (n=9) | 0.05144 | 0.007535 | 0.002512 | 0.05389 | 0.003689 | 0.001230 | 0.02111^@#^ | 0.005904 | 0.001968 | 0.06333^@$^ | 0.004301 | 0.001434 |

- Statistical analysis was done using ANOVA followed by Tukey’s as a post hoc test The differences were considered significant at P < 0.05 as compared to (@) the control, (#) ALO, and ($) RTBI groups. ALO: alogliptin; ns: non-significant; RTBI: repetitive traumatic brain injury; RTBI+ALO: repetitive traumatic brain injury post-treated with alogliptin orally for seven days.

**2-Descriptive statistics of the forced swimming test**

|  | Control | | | ALO | | | RTBI | | | RTBI+ALO | | |
| --- | --- | --- | --- | --- | --- | --- | --- | --- | --- | --- | --- | --- |
|  | **Mean** | **SD** | **SEM** | **Mean** | **SD** | **SEM** | **Mean** | **SD** | **SEM** | **Mean** | **SD** | **SEM** |
| Immobility time (n=9) | 0.8889 | 0.254 | 0.0514 | 0.8889 | 0.344 | 0.0547 | 16.89^@#^ | 2.667 | 0.8889 | 1.333^$^ | 0.532 | 0.0774 |
| Climbing time  (n=9) | 190.9 | 24.9 | 8.301 | 165.3 | 21.98 | 7.328 | 72.44^@#^ | 16.44 | 5.480 | 152.6^@$^ | 23.44 | 7.812 |

- Statistical analysis was done using ANOVA followed by Tukey’s as a post hoc test The differences were considered significant at P < 0.05 as compared to (@) the control, (#) ALO, and ($) RTBI groups. ALO: alogliptin; ns: non-significant; RTBI: repetitive traumatic brain injury; RTBI+ALO: repetitive traumatic brain injury post-treated with alogliptin orally for seven days.
- **Descriptive statistics of Immunohistochemistry**

**1- Descriptive statistics of Nrf2 and HO-1 in Fig.6**

|  | Control | | | RTBI | | | RTBI+ALO | | |
| --- | --- | --- | --- | --- | --- | --- | --- | --- | --- |
|  | **Mean** | **SD** | **SEM** | **Mean** | **SD** | **SEM** | **Mean** | **SD** | **SEM** |
| Nrf2  (n=3) | 8.792 | 0.4321 | 0.1932 | 1.170^@^ | 0.08631 | 0.0386 | 7.042^@#^ | 0.5856 | 0.2619 |
| HO-1  (n=3) | 7.694 | 0.4521 | 0.2022 | 2.090^@^ | 0.4006 | 0.1791 | 5.212^@#^ | 0.3432 | 0.1535 |

The differences were considered statistically significant at P < 0.05 as compared to the (@) Control and (#) RTBI groups. Nrf2: Nuclear factor erythroid 2-related factor 2; HO-1: heme oxygenase-1; RTBI: repetitive traumatic brain injury; RTBI+ALO: repetitive traumatic brain injury post treated with alogliptin orally for seven days.

|  | Control | | | RTBI | | | RTBI+ALO | | |
| --- | --- | --- | --- | --- | --- | --- | --- | --- | --- |
|  | **Mean** | **SD** | **SEM** | **Mean** | **SD** | **SEM** | **Mean** | **SD** | **SEM** |
| TNF-α  (n=3) | 0.7400 | 0.3187 | 0.1425 | 6.830^@^ | 1.059 | 0.4737 | 4.052^@#^ | 0.5393 | 0.2412 |
| NF-κB  (n=3) | 0.344 | 0.1552 | 0.06940 | 5.414^@^ | 0.7657 | 0.3424 | 2.582^@#^ | 0.3825 | 0.1710 |

**2- Descriptive statistics of TNF-α and NF-κB in Fig.7**

The differences were considered statistically significant at P < 0.05 as compared to the (@) Control and (#) RTBI groups. TNF-α: Tumor necrosis factor alpha; NF-қB: nuclear factor-kappa B; RTBI: repetitive traumatic brain injury; RTBI+ALO: repetitive traumatic brain injury post treated with alogliptin orally for seven days.

|  | Control | | | RTBI | | | RTBI+ALO | | |
| --- | --- | --- | --- | --- | --- | --- | --- | --- | --- |
|  | **Mean** | **SD** | **SEM** | **Mean** | **SD** | **SEM** | **Mean** | **SD** | **SEM** |
| Aβ  (n=6) | 137 | 8.32 | 3.396 | 229.1^@^ | 14.78 | 6.034 | 144.3^#^ | 17.72 | 7.233 |
| Tau  (n=6) | 0.6617 | 0.02787 | 0.01138 | 1.013^@^ | 0.07062 | 0.02883 | 0.7233^#^ | 0.02251 | 0.009189 |
| BDNF  (n=6) | 0.9467 | 0.03141 | 0.01282 | 0.5417^@^ | 0.03971 | 0.01621 | 0.9200^#^ | 0.03162 | 0.01291 |
| TrKB  (n=6) | 173.5 | 7.147 | 2.918 | 228.6^@^ | 24.67 | 10.07 | 373.5^@#^ | 48.94 | 19.98 |

- **Descriptive statistics of ELISA parameters**

**1- Descriptive statistics of Aβ, Tau, BDNF and TrKB in Fig.5**

- Statistical analysis was done using ANOVA followed by Tukey’s as a post hoc test. The differences were considered significant at P < 0.05 as compared to the (@) Control and (#) RTBI groups. Aβ: β‐amyloid; BDNF: Brain-derived neurotrophic factor; TrKB: tropomyosin receptor kinase B; RTBI: repetitive traumatic brain injury; RTBI+ALO: repetitive traumatic brain injury post-treated with alogliptin orally for seven days.

**2- Descriptive statistics of ATF6 and GRP78 in Fig.8**

|  | Control | | | RTBI | | | RTBI+ALO | | |
| --- | --- | --- | --- | --- | --- | --- | --- | --- | --- |
|  | **Mean** | **SD** | **SEM** | **Mean** | **SD** | **SEM** | **Mean** | **SD** | **SEM** |
| ATF6  (n=5) | 554.3 | 53.89 | 24.10 | 747.4^@^ | 80.66 | 36.07 | 527.7^#^ | 57.84 | 25.87 |
| GRP78  (n=5) | 0.8120 | 0.06870 | 0.03072 | 1.024^@^ | 0.05413 | 0.02421 | 0.7460^#^ | 0.1119 | 0.05006 |

Statistical analysis was done using ANOVA followed by Tukey’s as a post hoc test. The differences were considered statistically significant at P < 0.05 as compared to the (@) Control and (#) RTBI groups. GRP78: glucose-regulated protein 78; ATF6: activating transcription factor 6; RTBI: repetitive traumatic brain injury; RTBI+ALO: repetitive traumatic brain injury post-treated with alogliptin orally for seven days.

- **Descriptive statistics of miRNA-322 and miRNA-125b in Fig. 8**

|  | Control | | | RTBI | | | RTBI+ALO | | |
| --- | --- | --- | --- | --- | --- | --- | --- | --- | --- |
|  | **Mean** | **SD** | **SEM** | **Mean** | **SD** | **SEM** | **Mean** | **SD** | **SEM** |
| miRNA-322  (n=5) | 1 | 0 | 0.000 | 0.5861^@^ | 0.1259 | 0.05630 | 3.355^@#^ | 0.2060 | 0.09211 |
| miRNA-125b (n=5) | 1 | 0 | 0.000 | 12.83^@^ | 0.7272 | 0.3252 | 7.098^@#^ | 0.5441 | 0.2433 |

Statistical analysis was done using ANOVA followed by Tukey’s as a post hoc test. The differences were considered statistically significant at P < 0.05 as compared to the (@) Control and (#) RTBI groups. RTBI: repetitive traumatic brain injury; RTBI+ALO: repetitive traumatic brain injury post-treated with alogliptin orally for seven days.

- **Effect of ALO on brain morphology and cerebral cortex histoarchitecture after RTBI induction**


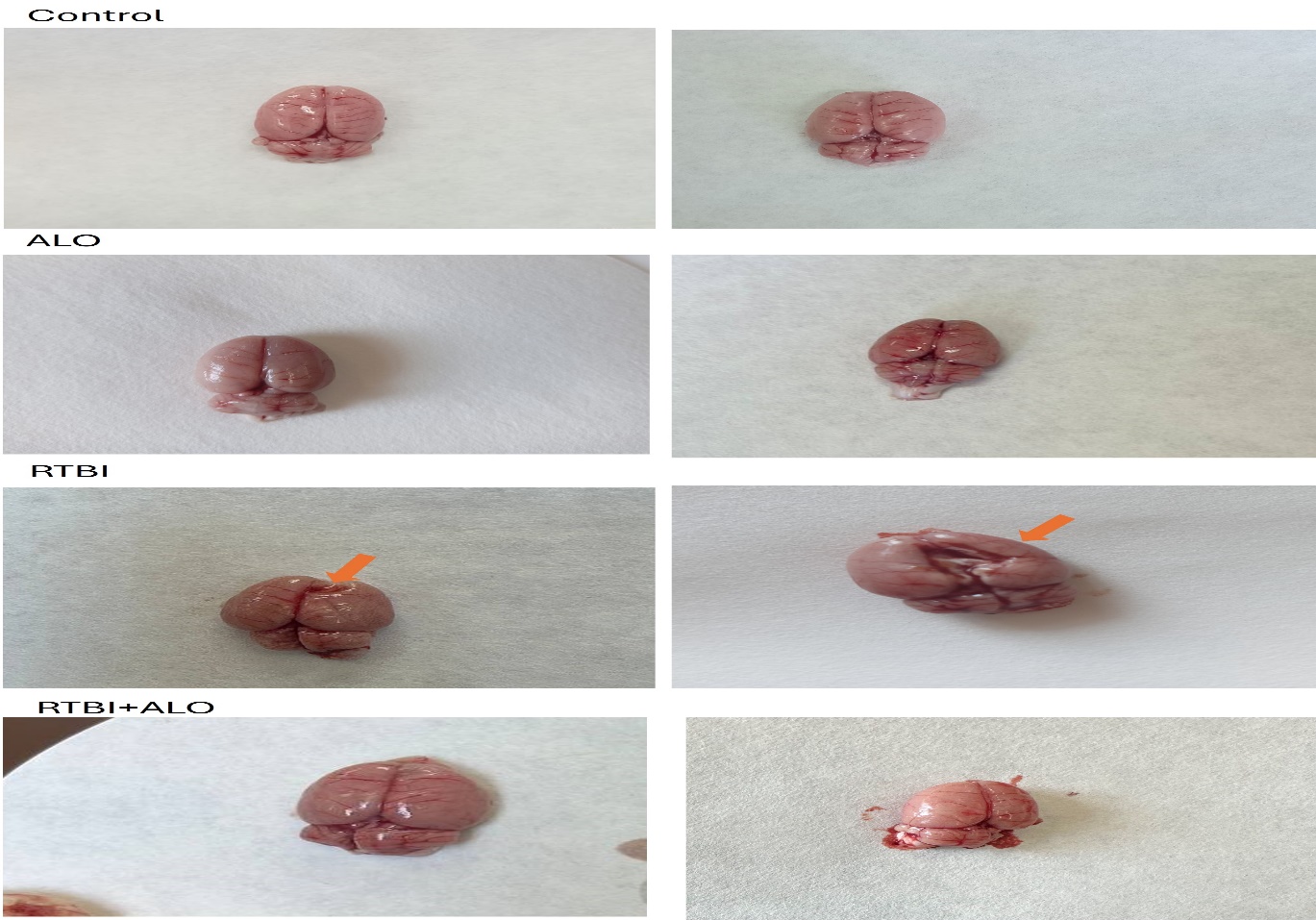


Impact of ALO on brain morphological appearance after RTBI induction. ALO: alogliptin; RTBI: repetitive traumatic brain injury; RTBI+ALO: repetitive traumatic brain injury post-treated with alogliptin orally for seven days; orange arrow: impact of five repetitive hits induced by weight drop model on the right interior frontal area.

**
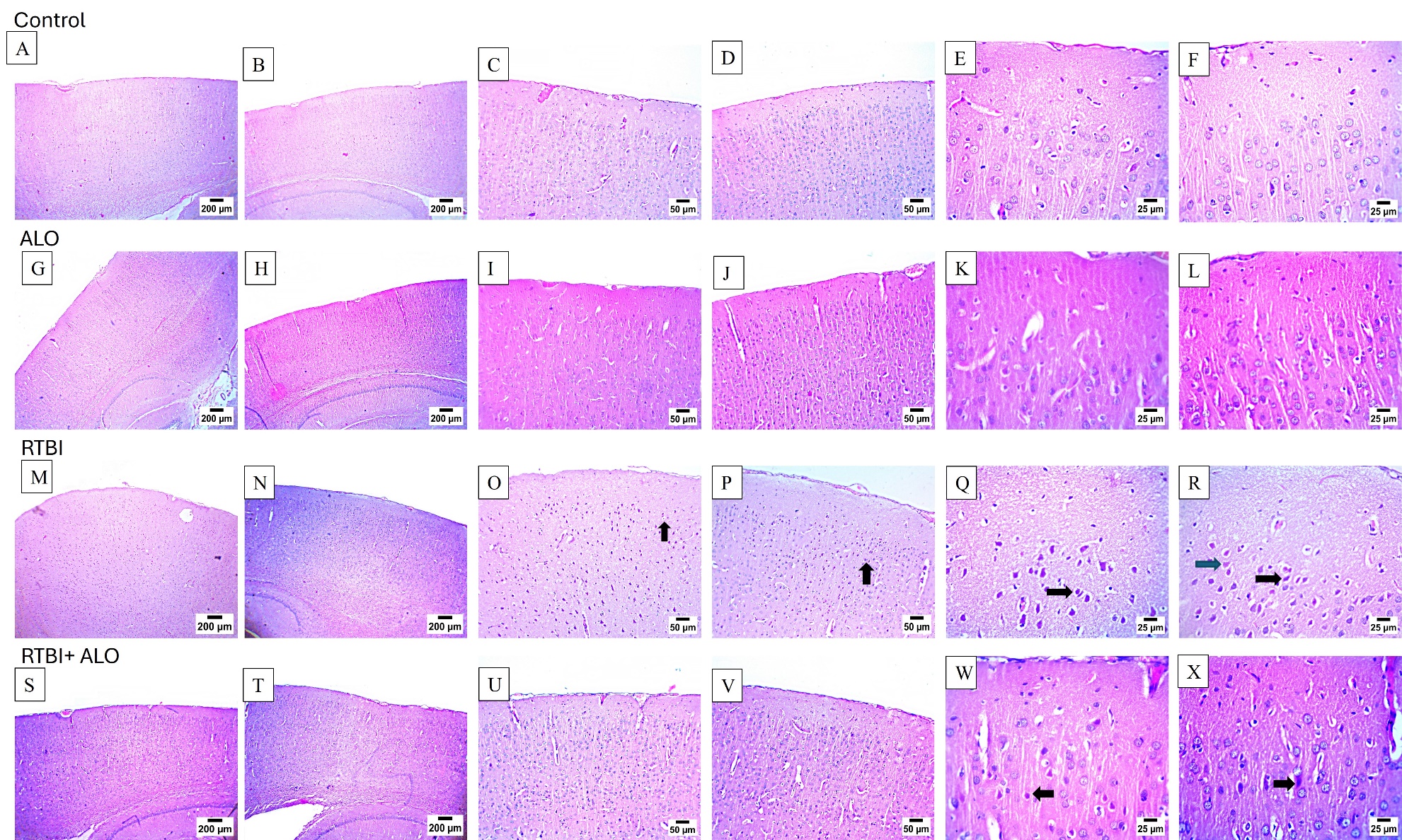
**

Representative photomicrographs of H&E-stained cerebral cortex of rats. Control (A, B, C, D, E, and F) & alogliptin (G, H, I, J, K, and L) groups show normal cerebral cortex. The RTBI (M, N, O, P, Q, and R) group shows a high number of degenerated and shrunken neurons with pyknotic nuclei (black arrow) in addition to a few numbers of necrotic cells (blue arrow). The RTBI+ alogliptin (S, T, U, V, W, and X) group shows a few numbers of degenerated and shrunken neurons with pyknotic nuclei (black arrow). ALO: alogliptin; RTBI: repetitive traumatic brain injury; RTBI+ALO: repetitive traumatic brain injury post-treated with alogliptin orally for seven days.

- **Effect of ALO on the levels of TNF-α and NF-κB after RTBI induction**

**
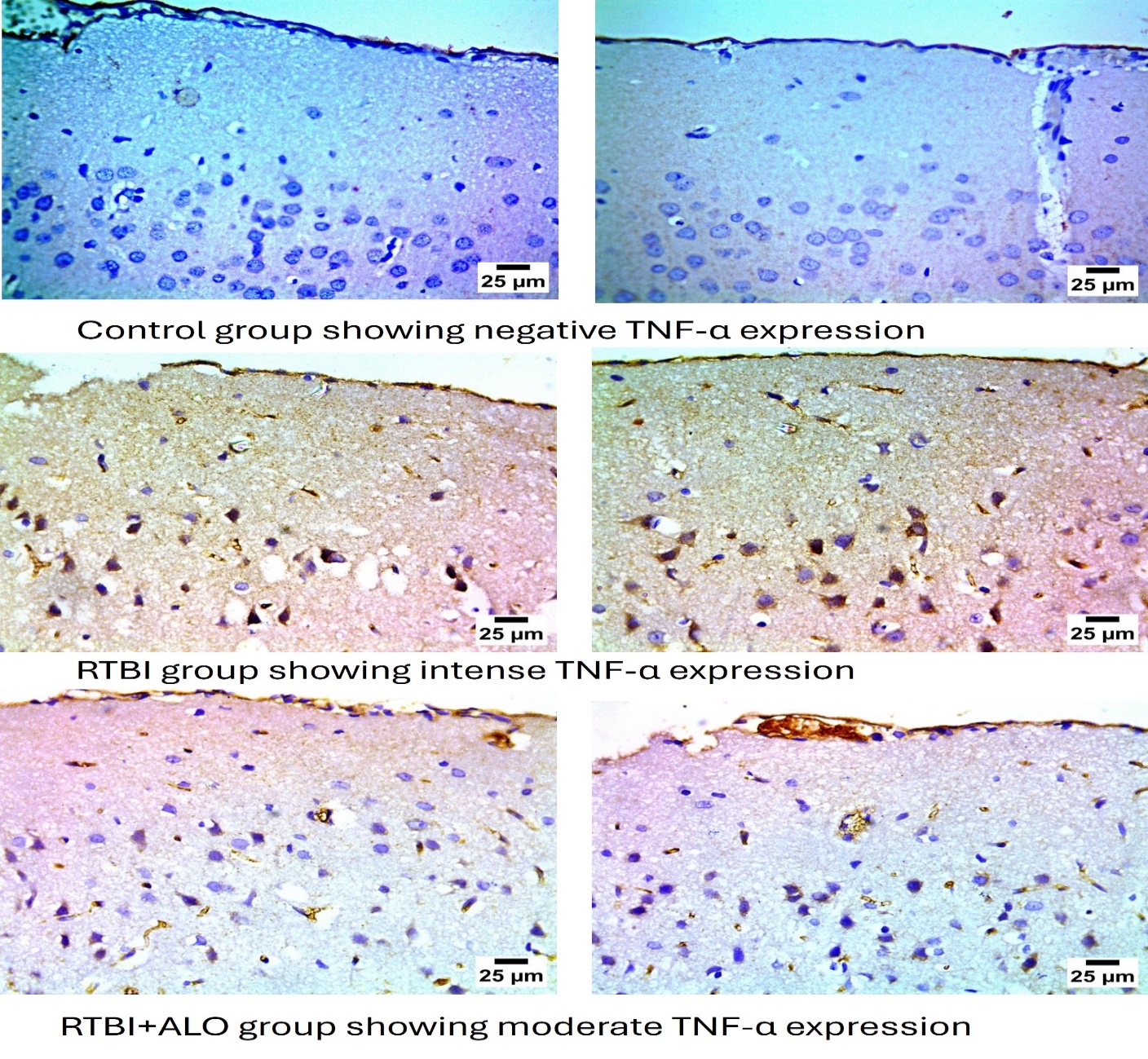
**

**
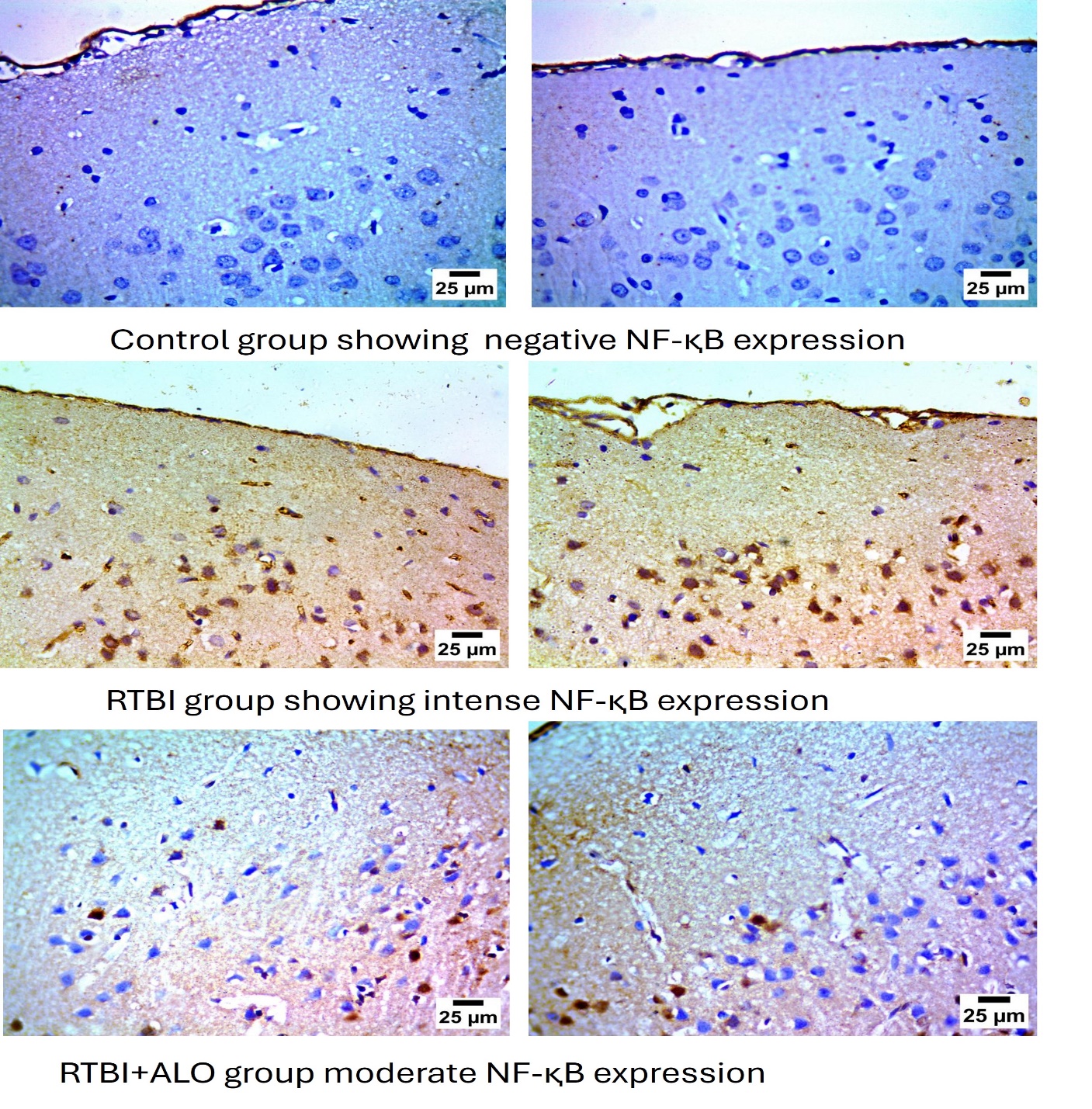
**

- **Effect of ALO on Nrf2 and HO-1 expression after RTBI induction**

**
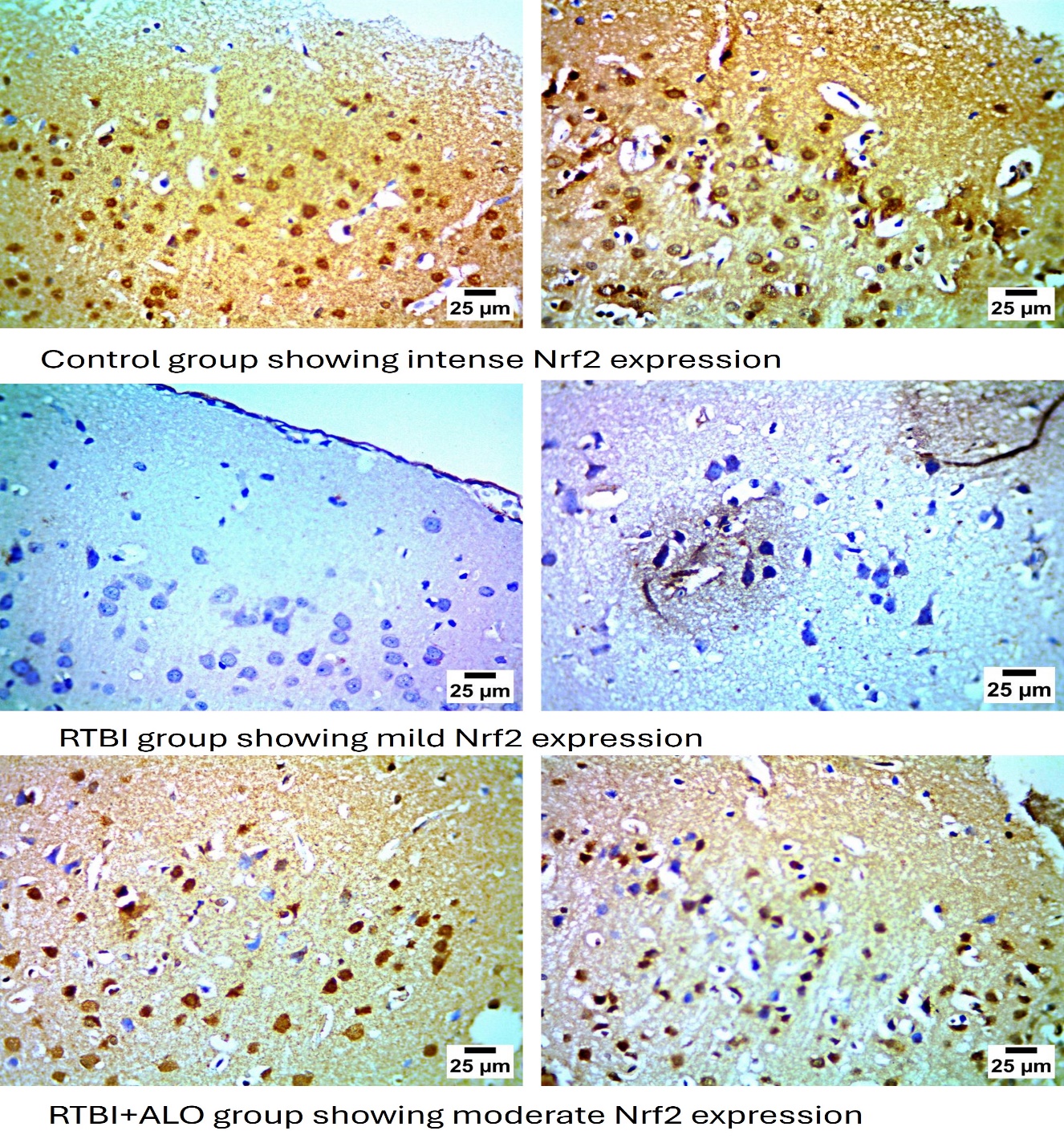
**


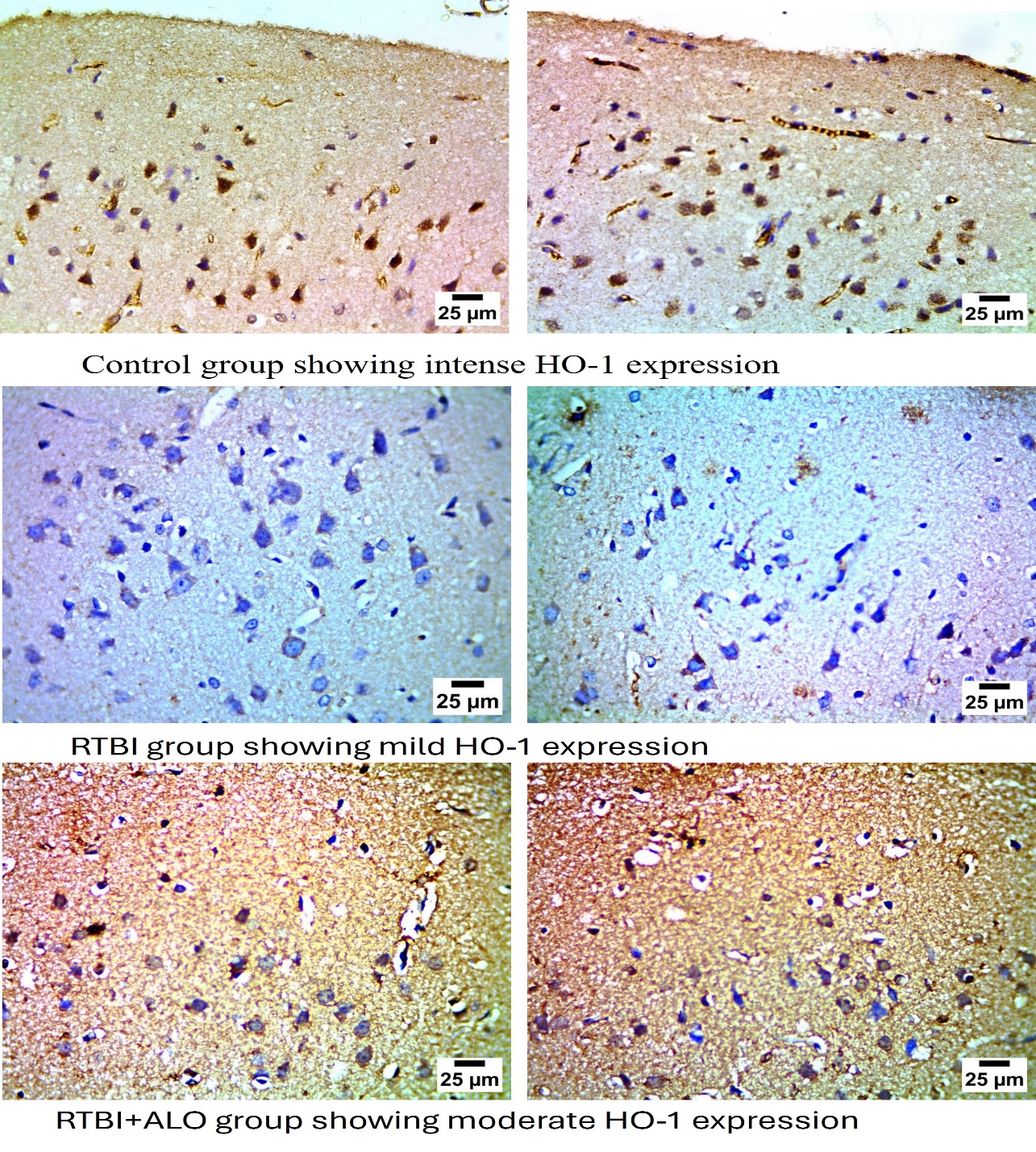


- **Cycling conditions for SYBR green real time PCR according to Quantitect SYBR green PCR kit**

| **Gene** | **Reverse transcription** | **Primary**  **denaturation** | **Amplification (40 cycles)** | | | **Dissociation curve**  **(1 cycle)** | | |
| --- | --- | --- | --- | --- | --- | --- | --- | --- |
|  |  |  | **Secondary denaturation** | **Annealing**  **(Optics on)** | **Extension** | **Secondary denaturation** | **Annealing** | **Final denaturation** |
| **U6**  **(housekeeping)** | 50˚C  30 min. | 94˚C  15 min. | 94˚C  15 sec. | 60˚C  30 sec. | 72˚C  30 sec. | 94˚C  1 min. | 60˚C  1 min. | 94˚C  1 min. |
| **MiRNA-322** | 50˚C  30 min. | 94˚C  15 min. | 94˚C  15 sec. | 60˚C  30 sec. | 72˚C  30 sec. | 94˚C  1 min. | 60˚C  1 min. | 94˚C  1 min. |
| **MiRNA-125b** | 50˚C  30 min. | 94˚C  15 min. | 94˚C  15 sec. | 50˚C  30 sec. | 72˚C  30 sec. | 94˚C  1 min. | 50˚C  1 min. | 94˚C  1 min. |

- **Effect of ALO on abnormal Aβ aggregation after RTBI induction**

A

- **Effect of ALO on abnormal Tau aggregation after RTBI induction**

B

- **Effect of ALO on cortical contents of BDNF after RTBI induction**

C

- **Effect of ALO on cortical contents of TrKB after RTBI induction**

D

- **Effect of ALO on miRNA-322 gene expression**


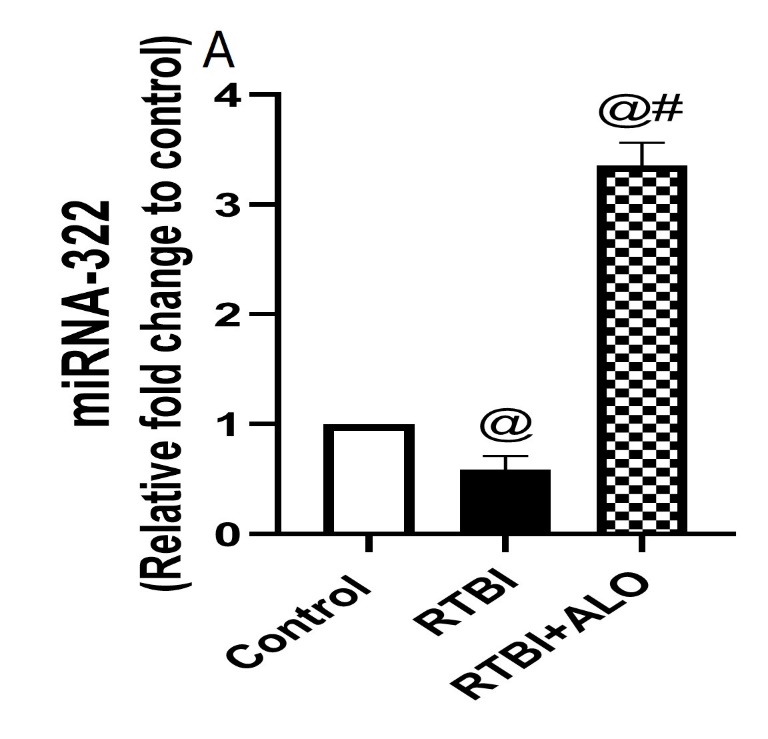


- **Effect of ALO on miRNA-125b gene expression**


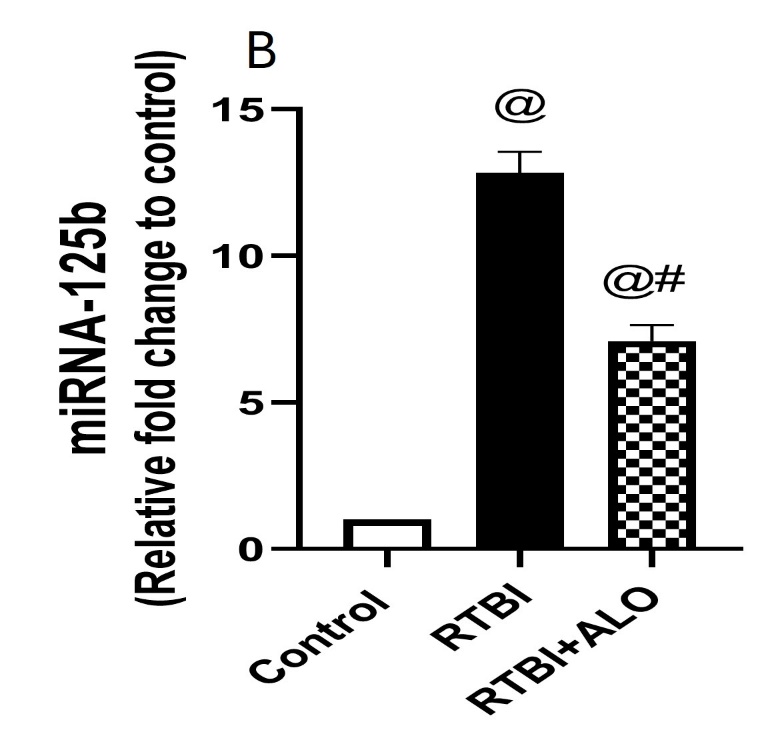


- **Effect of ALO on cortical contents of GRP78 after RTBI induction**


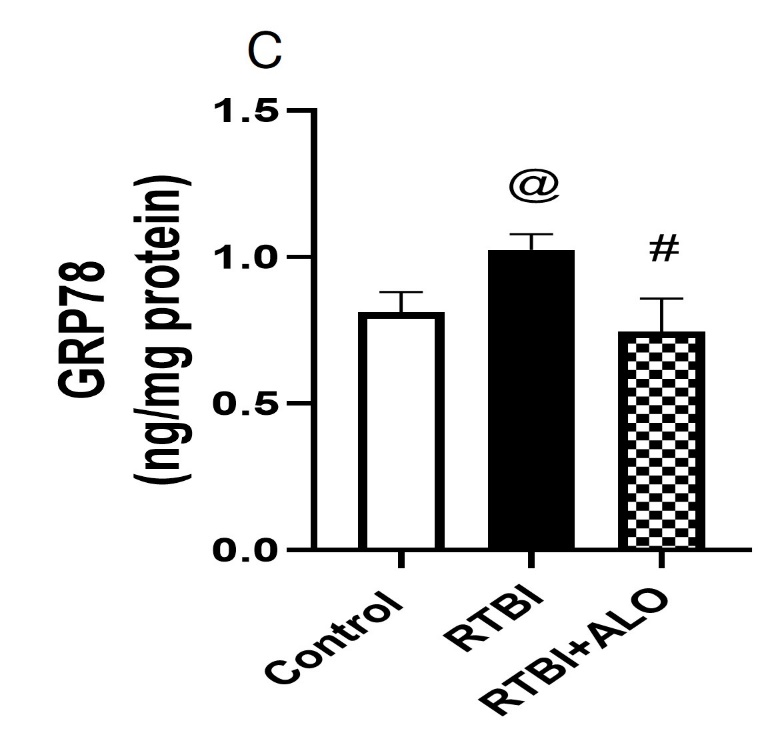


- **Effect of ALO on cortical contents of ATF6 after RTBI induction**


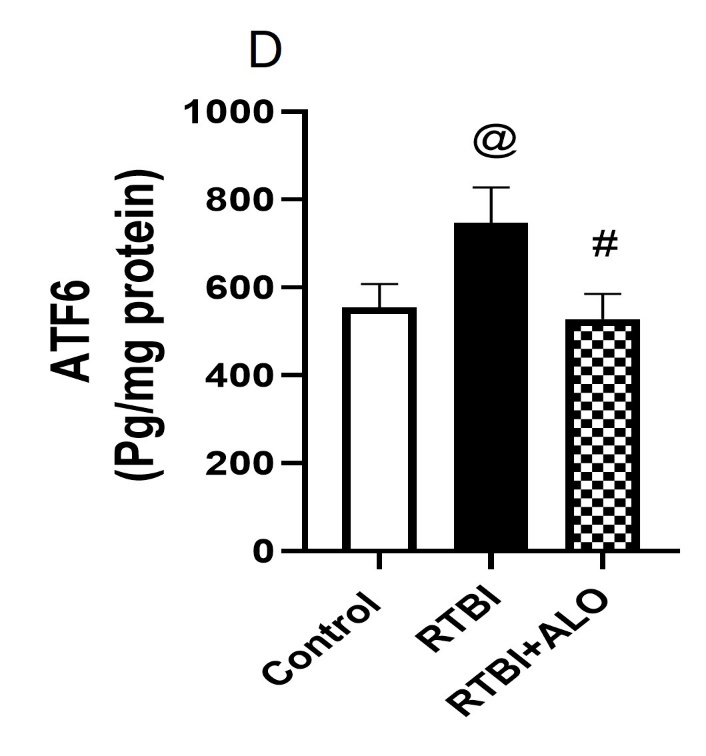

Supplement: Supplementary file 1 — Supplementary Material 1 (DOCX 5.78 MB) [file 11481_2025_10271_MOESM1_ESM.docx]
